# Achieving lysozyme functionalization in PDADMAC–NaPSS saloplastics through salt annealing†

**DOI:** 10.1039/d4ra04986a

**Published:** 2024-10-18

**Authors:** Jiaying Li, Lijie Li, Saskia Lindhoud

**Affiliations:** a Department of Molecules and Materials, MESA+ Institute for Nanotechnology, University of Twente, Faculty of Science and Technology P. O. Box 217 7500 AE Enschede The Netherlands s.lindhoud@utwente.nl

## Abstract

Hot-pressed saloplastics are dense and transparent polyelectrolyte complex materials governed by ionic crosslinking. Such plastics have several advantages, for example, salt water processibility and recyclability. Here, we demonstrate a simple but effective post-treatment method to incorporate lysozyme as a biocatalytic component into the hot-pressed saloplastics. Changes in salt concentration can be used for annealing and curing the saloplastics, where the temporary opening allows for lysozyme loading. This process was carefully examined by two different routes and the salt concentrations and incubation times were varied systematically. Optimised saloplastics showed an enzymatic activity against *Micrococcus lysodeikticus* of 4.44 ± 0.37 U cm^−2^ and remained partially active (∼72% activity preserved) after 7 days. This approach opens new routes to incorporate enzymes or other biological functionality into saloplastics which is difficult to achieve for conventional plastics.

## Abstract 2

Salt treatments enable the functionalization of hot-pressed saloplastics with lysozyme.

## Graphical abstract

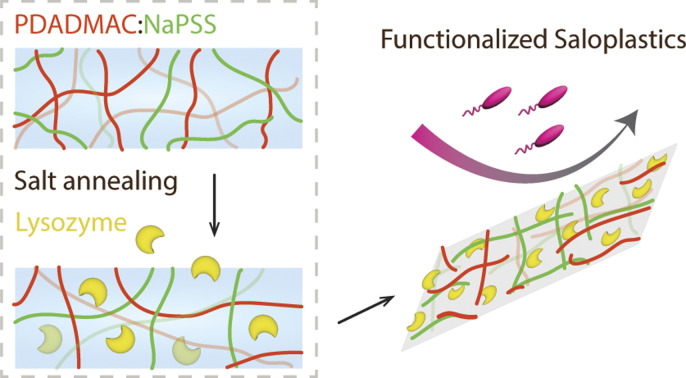

## 1 Introduction

Plastics are inexpensive, lightweight, and easily processable materials that can be found almost everywhere, from food packaging and furniture, to cars.^1^ They have become dominant as a material because of these advantages, and slowly replaced materials derived from natural sources such as paper and wood. Modern society heavily relies on plastics and its annual production has reached ∼460 million tonnes in 2019 and is expected to reach 1.23 billion tonnes in 2060.^2,3^ Packaging, especially food packaging, is the main application of plastics and contributes to nearly 40% of the production.^4^ Moreover, most of these food packaging plastics are for single use.^5^ As a result of difficult separations, lack of cost-efficiency, and technical limitations, the recycling rate of plastics was only ∼9% (2015).^2,6–8^ The majority of plastic wastes are incinerated or landfilled, causing environmental and potential health problems such as air pollution, water pollution, and microplastics in food.^9–13^ One less obvious but relevant application of plastics is for lab use, especially microbiological labs, such as Petri dishes, pipette tips, and various tubes.^14^ In 2014, the total estimated value of plastic wastes generated from worldwide biological, medical, and agricultural labs was ∼5.5 million tonnes.^15^ The science community has recognized this issue and has pointed out that we should reduce the amount of usage of single-use plastics, find other alternatives, and better reuse/recycle plastics during research.^15–19^

Researchers have been investigating more sustainable alternatives. One approach is to use biobased and/or biodegradable polymers, for example, polylactic acid, cellulose, and bio-polyethylene.^20,21^ Another approach is to utilize reversible mechanisms, such as dynamic covalent bond (DCB),^22,23^ hydrogen bonding,^24–26^ and electrostatic interaction.^27,28^ Polyelectrolytes are charged polymers that can form complexes *via* opposite charges and the driving force is entropy due to the release of counterions.^29^ Naturally, salt can act as a doping agent to tune the ionic crosslinking of the complexes and potassium bromide (KBr) is one of the most commonly used salts which has shown an effective doping effect on polyelectrolyte complexes (PECs).^30–32^ The term “saloplastics” is given to describe these PECs, similar to the effect of temperature on thermoplastics.^28^ Krishna B. *et al.* have developed a hot-pressed method to produce dense transparent saloplastics with controlled thicknesses.^33^ Processable poly(diallyl dimethyl ammonium chloride)–sodium polystyrene sulfonate (PDADMAC–NaPSS) complexes were first formed by tuning the salt strength, then they were hot-pressed by tuning the temperature and pressure. This approach opens up a new door to process PECs and provides a new platform to incorporate functionalities.

One relevant functionality is antimicrobial/antibacterial property. Plastics are not intrinsically antimicrobial thus microbial contaminations can cause health problems in food packaging or biomedical applications.^34,35^ Antimicrobial property is also essential for microbiological labs where cross contaminations should be avoided for both research and health reasons.^36,37^ The most common method to enable antimicrobial functionality of plastics is by blending in antimicrobial agents, such as metal ions (copper, silver) or biocides (quaternary ammonium compounds (QACs), chlorine-releasing agents).^38–40^ Lysozyme, commonly derived from chicken egg white, is an antibacterial enzyme that effectively attacks Gram-positive bacteria and works less sufficiently for Gram-negative bacteria.^41,42^ It has been shown that lysozyme can be taken up by complexes consisting of different oppositely charged polyelectrolyte pairs^43^ and this protein can be incorporated in polyelectrolyte complex membranes formed by aqueous phase separation.^44,45^ To detect its enzymatic activity, *Micrococcus lysodeikticus* (Gram positive) is usually used.^46^ Lysozyme is easily accessible and has been investigated as an antibacterial agent incorporated in polyvinyl alcohol films,^47^ chitosan films,^48^ wool fabrics,^49^ and polyelectrolyte complex membranes.^44^ To integrate lysozyme into the saloplastics, salt is again utilized to allow chain rearrangements and weaken the ionic interaction.^50,51^ This salt annealing approach makes it possible to incorporate bio-contents into saloplastics which is usually not possible with conventional plastics where heat is involved for processing.

In this work, we aim to prepare lysozyme-functionalized saloplastics. We first prepared hot-pressed PDADMAC–NaPSS films *via* their bulk complexes. Followed by a post-treatment step, ionic crosslinks were broken or loosened by adding different concentrations of KBr. Small pores were formed that allow for the incorporation of lysozyme. After reducing the KBr concentration, the strong ionic network was restored and lysozyme was trapped inside the complex. We further examined the lysozyme activity and stability to prove its potential to be applied as antibacterial plastics.

## 2 Experimental section

### 2.1. Materials

Poly(sodium 4-styrenesulfonate) (NaPSS, *M*_w_ 1000k g mol^−1^, powder), poly(diallyldimethylammonium chloride) (PDADMAC, average *M*_w_ 400k–500k g mol^−1^, 20 wt% in water), potassium bromide (KBr, ACS reagent, ≥99.0%), glycerol solution (83.5–89.5% (T)), lysozyme from chicken egg white (L6876, lyophilized powder, protein ≥ 90%, ≥40 000 units per mg protein), sodium phosphate monobasic (ReagentPlus®, ≥99.0%), sodium phosphate dibasic (Puriss. p.a., ACS reagent, anhydrous, ≥99.0% (T)), and *Micrococcus lysodeikticus* (M3770) were purchased from Sigma-Aldrich (The Netherlands). All water used was deionized water (Milli-Q®, Merck, The Netherlands). All chemicals were used as received without further purification.

### 2.2. Preparation of polyelectrolyte complexes

The concentration of received PDADMAC was found to be lower than 20 wt% indicated by the manufacturer. To obtain the correct concentration, PDADMAC was first dried in the oven at 80 °C for ∼4 h, then the obtained solid was stored at 30 °C under vacuum. To prepare PDADMAC–NaPSS complexes, single polyelectrolyte 2 wt% stock solutions were first prepared with 250 mM KBr in each. Then, different PDADMAC : NaPSS mixtures were prepared at a mixing monomer ratio of 1 : 1, 1 : 1.5, 1 : 2, and 1 : 2.5 using *M*_NaSS_ = 206.2 g mol^−1^ and *M*_DADMAC_ = 161.7 g mol^−1^. These two solutions were poured simultaneously into a beaker and stirred for 2 h. After obtaining the complex, it was cut into small pieces and washed thoroughly with water. Then, the complex was dried in the oven at 80 °C for 4 h. At last, the complex was ground to ∼2 mm particles using a coffee bean grinder and stored at 30 °C under vacuum.

### 2.3. Hot-pressing

Delrin® (Dupond) plates were used as the mould for hot-pressing. A spacer of thickness ∼0.183 mm was made by PTFE coated fiberglass (Lubriglas®-CHAP-1540, Reichelt Chemietechnik GmbH + Co, Germany). It was glued to the edges of the bottom plate with small outlets to allow extra water and complex escape. The area for pressing was ∼8 cm × 11 cm. An FV20R Rolling Driptech Rosin Press (FlashVape, Canada) was used for hot-pressing. The dry complex was first soaked in a 0.5 M KBr solution for ∼48 h and ∼4.5 g wet complex was used for each press. The wet complex was placed in the center of the bottom plate. After closing the plates, the mould was loaded between the heating plates of the hot-press. The temperature was heated to 90 °C gradually (2 °C min^−1^) and stayed at 90 °C for ∼40 min. Afterwards, 200 bar pressure was applied and maintained for ∼15 min. At last, the plates were cooled back to room temperature and the pressure was removed.

### 2.4. Pore formation and closure (salt annealing + water soaking and curing)

**Fig. 1:**
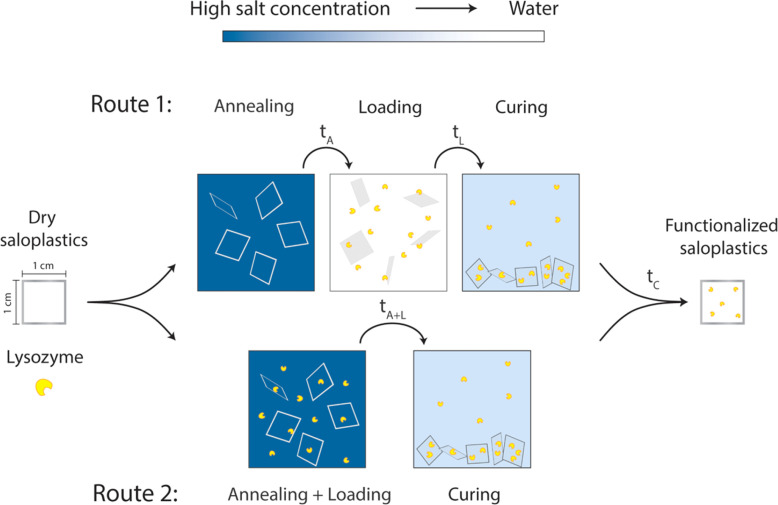
Proposed two routes to prepare lysozyme-functionalized saloplastics by utilizing different salt concentrations.

As discussed in the introduction, KBr is utilized to modify the structure of saloplastics to load lysozyme. Before lysozyme loading, first the response of saloplastics to different annealing and curing procedures *i.e.*, opening and closing of the pores, was determined. To find the suitable KBr concentrations for opening and closing the structure, saloplastics at a PDADMAC : NaPSS monomer mixing ratio of 1 : 1 were used. Samples were cut into 1 cm × 1 cm pieces. Annealing was first performed in different KBr concentrations (0, 0.1, 0.3, 0.5, 0.7, and 1 M) for different periods of time (1 min, 5 min, 15 min, 30 min, 1 h, and 2 h). From this experiment, the annealing time for lysozyme functionalization would be determined which is shown as *t*_A_ in Fig. 1. After salt annealing, saloplastics were immersed in water overnight (∼12 h). The abrupt alteration in salinity levels resulted in the formation of pores. Since pores were available for lysozyme uptake, this time would also be the loading step *t*_L_ in Fig. 1. At this stage, the structural change was the main focus thus no lysozyme was added yet. The increase in porosity would increase the light scattering thus the films appeared white.^52^ The whiteness of each sample was determined using a whiteness extraction method using pictures and ImageJ (Fig. S1 and eqn (S1)†). After salt annealing and water soaking, it is also important to check whether salt can cure the structure to close the pores. Similarly, chosen samples were cured in different KBr concentrations (0.1, 0.3, 0.5, 0.7, and 1 M, no higher than annealing KBr concentration) for different periods of time (1 min, 5 min, 15 min, 30 min, 1 h, and 2 h). From this experiment, the curing time would be determined which is shown as *t*_C_ in Fig. 1. In the end, their whiteness was checked using the same method to examine whether the films can return to an almost transparent state, which suggests a dense non-porous structure.

The morphology and structure of these saloplastics before/after salt annealing/curing were examined by a scanning electron microscope (SEM, JSM-6010LA, Japan). In a previous study, PDADMAC–PSS membranes were pre-soaked in 2-propanol to prevent the pore structure from collapsing during drying.^52^ Instead, to be able to maintain the same KBr concentration, the samples were soaked in a 20 wt% glycerol + KBr solution for 4 h. After drying inside the fume hood, the dry samples were stored further at 30 °C under vacuum overnight. All samples were coated with a 5 nm Pt/Pd layer to induce conductivity (Quorum Q150T ES, Quorum Technologies, Ltd, UK). The diameters of some pores were labelled by ImageJ.

### 2.5. Lysozyme incorporation

To determine a suitable concentration for loading, the saloplastics were cut into 1 cm^2^ pieces and soaked in a 1 M KBr solution for 2 h. After salt annealing, these pieces were immersed in different concentrations of lysozyme solutions. The studied range was 5, 10, 25, and 50 mg L^−1^ 10 pieces were first stirred for 4 h in a 20 mL lysozyme solution then they were submerged overnight at 4 °C. After taking out the saloplastics, UV-Vis measurements of the solutions were conducted using a PerkinElmer Lambda 850, US. The absorbance at 281.5 nm is a characteristic peak for lysozyme. By measuring how much lysozyme was left in the solutions, a suitable excess lysozyme concentration was selected for the loading step.

The loading of lysozyme was conducted *via* two routes as shown in Fig. 1: (1) firstly, salt annealing was performed by soaking saloplastics in a KBr solution. For the second loading step, a lysozyme–water solution was used to induce a concentration difference, thereby creating micropores that permit the penetration of lysozyme into the saloplastic films. Finally, samples were cured in a KBr solution with lower concentration than salt annealing to close the pores and trap the lysozyme within the saloplastics. (2) Lysozyme was added to the annealing solutions to combine the annealing and loading steps, then followed by the same curing step. For all curing steps, to avoid lysozyme escaping, the same concentration of lysozyme was added into the curing solutions. The suitable KBr concentration and duration for each step were decided later after obtaining the salt annealing and curing results. The experimental details for both routes are given in Table 1 and the discussion can be found in Section 3.2.3. The code names of each sample (A: annealing, L: Loading, C: curing + different KBr concentrations) are also summarized in Table 1.

Sample preparation following route 1 or 2 and their corresponding names. (1) Salt annealing, lysozyme loading, and curing; (2) salt annealing + lysozyme loading, and curing

**Table:** 

Route 1	Salt annealing (*t*_A_ = 1 h)	Lysozyme loading (*t*_L_ = overnight)	Curing (*t*_C_ = 1 h)	Sample names
KBr (M)	0	0	0	A0 L0 C0
	0.5		0.3	A0 L0 C0.3
	1			A0.5 L0 C0
				A0.5 L0 C0.3
				A1 L0 C0
				A1 L0 C0.3

**Table:** 

Route 2	Salt annealing + lysozyme loading (*t*_A+L_ = overnight)	Curing (*t*_C_ = 1 h)	Sample names
KBr (M)	0	0	AL0 C0
	0.5	0.3	AL0 C0.3
	1		AL0.5 C0
			AL0.5 C0.3
			AL1 C0
			AL1 C0.3

### 2.6. Enzymatic activity

The activity of lysozyme was measured by tracking the characteristic peak of *Micrococcus lysodeikticus* at 450 nm. A 0.15 g L^−1^ suspension of this bacteria was prepared in a 50 mM sodium phosphate buffer (PBS) at pH 6.2. To remove excess lysozyme attached on the surface, 1 cm^2^-sized pieces of saloplastics were soaked in 50 mL of KBr solution (same concentration as the curing step) for 5 min and then it was placed in a new KBr solution. This procedure was repeated 3 times. Between each step and upon measuring, the sample was rinsed with the same KBr solution on both sides and wiped gently with dust-free tissues. The sample was then submerged in 2.5 mL of the *Micrococcus lysodeikticus* suspension. The decrease in absorbance at 450 nm was tracked over time (*t* = 0, 1, 2, 3, 4, and 5 h). For comparison, blank saloplastics without lysozyme were measured. 100 μL free lysozyme solutions (5 mg L^−1^) or PBS solution in a 2.5 mL suspension were also measured. The activity of lysozyme (*U*) was calculated by eqn (1):1
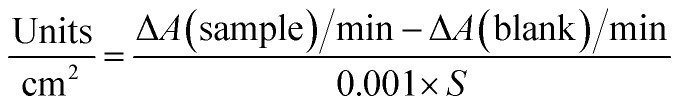
where Δ*A*(sample)/min and Δ*A*(blank)/min are the absorbance change at 450 nm per minute of the samples with and without lysozyme. 0.001 is pre-defined and *S* is the surface area (1 cm^2^).

For each data point, 3 samples were measured and the average results with standard deviations are reported. The activity of lysozyme in different samples was tested on the same day right after preparation (day 0). Samples with the best performance were stored in the final curing solution or dry conditions at 4 °C for 7 days to further investigate the lysozyme stability. All measurements were conducted at room temperature (∼21 °C).

To determine the leakage of lysozyme from the saloplastics over time, a piece of 1 cm^2^ saloplastics with or without lysozyme was stored in 2 mL water at 4 °C. At day 7, the enzymatic activity of the soaked saloplastics was tested and the supernatant was tested for leaked-out lysozyme by UV-Vis. The leakage behavior with the presence of KBr was also investigated by storing samples in 2 mL different concentrations (0.3, 0.5, and 1 M) of KBr solution. For comparison, free lysozyme solutions (10 mg L^−1^) stored in different KBr concentrations (0, 0.3, 0.5, 1 M) were also measured on day 7. For each data point, triplets were measured.

## 3 Results and discussion

The aim of our work is to study whether lysozyme can be taken up by saloplastics and whether the so obtained saloplastics exhibit antimicrobial activity. In order to achieve this, first we will discuss the preparation of the hot-pressed saloplastics, then the pore formation and closure of the pores need to be studied systematically. Here, we used two different routes that will first be discussed. The charge ratio between the polyelectrolytes could influence the uptake of the positively charged lysozyme, the effect of this ratio on lysozyme uptake was therefore studied. Finally, the uptake, leaking, and enzymatic activity also as a function of time will be considered.

### 3.1. Preparation of saloplastics

Saloplastics were prepared by modifying Krishna B. *et al.*'s recipe.^33^ Each step of the preparation was summarized in Fig. S2.† We chose PDADMAC and NaPSS (strong polycation and strong polyanion) because it is the most established system that has been systematically studied.^53^ Samples with different monomer mixing ratios were prepared to study the charge effect on lysozyme loading. Since lysozyme has an isoelectric point of 10–11 and is positively charged under the experimental conditions,^54–56^ it could be beneficial to prepare more negatively charged saloplastics by adding more NaPSS. For PDADMAC : NaPSS polyelectrolyte complexes, it is known that lysozyme uptake depends on the ratio between PDADMAC : NaPSS. Maximal lysozyme incorporation is observed when NaPSS is slightly in excess.^43^

The final thickness of saloplastics was measured by a micrometer and the average results are shown in Table 2. From the results, the overall thickness difference among different ratios was small. The water uptake (eqn (S2)†) of saloplastics at different ratios are also included in Table 2 and there was no significant difference. For all ratios, the water uptakes were close to what was reported in literature (∼40%).^57^

**Table:** Dry thicknesses and water uptakes of saloplastics at different PDADMAC : NaPSS ratios

PDADMAC : NaPSS	1 : 2.5	1 : 2	1 : 1.5	1 : 1
Thickness (μm)	97 ± 5	103 ± 4	111 ± 7	106 ± 3
Water uptake (%)	41.5 ± 0.6	42.8 ± 0.7	42.7 ± 0.4	45.4 ± 0.5

### 3.2. Effect of salt concentration

After obtaining the saloplastics, we can use a change in salt concentration to induce pore formation and close the pores again. As discussed in the introduction, salt plays an important role in determining the interaction strength between the oppositely charged polyelectrolytes. During the hot-pressing procedure, salt acts as a plasticizer to induce chain mobility. The final saloplastics consist of polyelectrolyte (PE) chains (PSS^−^ and PDADMA^+^) and residual salt ions (K^+^ and Br^−^). Increasing the salt concentration can lead to a shift to more extrinsic interactions (PE–counterion) than intrinsic interactions (PE–PE).^58,59^ As a result, the distance among PE chains increases until they fully dissociate into a solution.^60^ On the contrary, reducing the salt concentration can shift PECs from extrinsic to more intrinsic charges. When a large salt difference is presented, a porous structure can be induced when water gets expelled.^61^ This inversion process is similar to the preparation of a PEC membrane.^52,62,63^ This reversible structural change can thus be used to capture lysozyme. Salt concentration, as the most vital parameter, is further investigated by controlling both the annealing (opening of the pores) and curing (closing of the pores) steps.

Two different routes (Fig. 1) were proposed for the lysozyme encapsulation. Following route 1 (R1), the saloplastics were first soaked in a KBr solution for a certain period of time (*t*_A_) to increase the chain flexibility and free volume. Pores can be created by going from a high salt concentration to deionized water during the loading step (*t*_L_). These pores then became available to incorporate lysozyme. After curing (*t*_C_), the pores closed and lysozyme can be trapped in the matrix. For route 2 (R2), we combined the annealing and the loading step. With this combined step (*t*_A+L_), lysozyme was prefilled into the matrix. Followed by the same curing (*t*_C_), the distance among chains were decreased by reducing the salt concentration and resulted in trapping the prefilled lysozyme. For R2, lysozyme stability in the presence of salt would be examined first. The hydrodynamic radius of lysozyme is ∼1.1–2.9 nm.^64,65^ Thus, the created pores *via* R1 or R2 should be bigger than this to capture lysozyme. After curing, the distance should be smaller than this value to prevent lysozyme from escaping.

#### 3.2.1. Pore formation (annealing + water soaking)

Before loading the lysozyme, it is essential to study the structural changes induced by salt annealing and curing. Different salt concentrations were investigated to anneal the saloplastics. After immersion into the KBr solutions, the saloplastics expanded in size and gradually became more flexible. At a concentration of 1 M, 0.7 M, 0.5 M or 0.3 M, all films remained transparent, while at a concentration of 0.1 M, films turned temporarily white. This phenomenon indicates that the initial concentration of dry saloplastics was probably higher than 0.1 M because a salt-out effect was observed (Fig. S3†). After annealing, the saloplastics were immersed in water during a soaking step. In this step, pores were formed, giving the saloplastics a white appearance. These were the first two steps of R1 where we investigated the effect of pore formation on lysozyme capture.

**Fig. 2:**
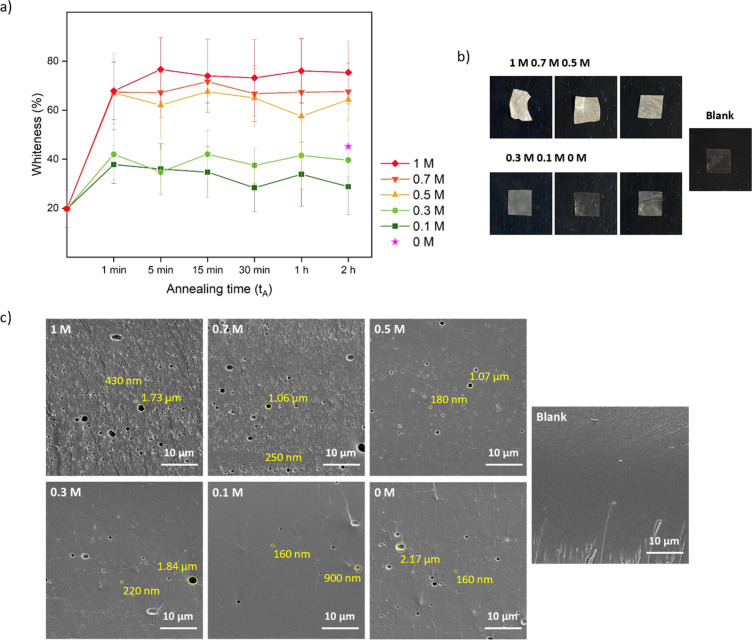
Pore formation induced by salt annealing and water soaking represents the first two steps of R1. (a) The whiteness change over time of different KBr concentration annealed samples. The whiteness change is summarized with standard deviations obtained from 3 replicates. (b) Pictures and (c) cross-sectional SEM images of 2 h-annealed samples after soaking in water overnight and the blank sample. These SEM images were taken from the middle area of the films. The diameters of some pores were measured and labelled in yellow.

One conventional method to determine the transparency of films is UV-Vis and it measures the absorbance within a visible wavelength range.^66^ Here, we developed a simple method (Fig. S1†) by extracting the whiteness information from pictures quickly taken while the saloplastics remained wet, preventing the collapse of the pores by drying. Moreover, instead of measuring one specific spot, colour information can be extracted down to every pixel which represents the inhomogeneity of the whole film. As shown in Fig. 2a, the final whiteness of the saloplastics which was induced from annealing in salt solutions, followed by immersion into water, highly depends on the KBr concentration. Here, films soaked in water overnight (∼12 h) were compared. The dry blank sample appeared transparent by eye, but still showed a whiteness ∼20%. Driven by the concentration differences, salt ions rushed out from the complex and left pores inside which caused the saloplastics to appear white. The diffusion of ions occurred fast that the saloplastics turned white at ∼1 min where further increasing the annealing time did not increase the overall whiteness. As the annealing concentration decreased, the films were overall less white. When performing the annealing in water, a similar value was observed as annealing in 0.3 M. The possible explanation could be the original KBr concentration of dry saloplastics was close to 0.3 M.

As discussed in Section 2.4, after salt annealing, samples were immersed in water overnight (∼12 h) to allow for salt ion releasing. The releasing speed also depends on the KBr concentration. For saloplastics annealed at 1 M (1 min annealing), it became white instantly when immersed in water, while for saloplastics annealed at 0.5 M (1 min annealing), the process slowed down and took ∼1 min (Videos included in the ESI†).

As shown in Fig. 2b, increasing the KBr concentration increased the film whiteness. The addition of KBr brought saloplastics from a solid state to a more coacervate-like state. At 1 M KBr, the films became flexible and sticky, thus when immersed in water, the fast salt diffusion “quenched” the films and kept the wavy structures. This phenomenon became less profound when reducing the concentration. From the cross-sectional SEM images, different levels of porosity *vs.* concentration were observed (Fig. 2c), similar to what has been observed in literature.^67,68^ For capturing the lysozyme, it is ideal to have homogeneously distributed small pores that match with the size of lysozyme. When increasing the concentration, pores were indeed generated which is beneficial for the encapsulation. However, these pores mainly range from few hundred nm up to 2 μm as shown in Fig. 2c. These large pores may be formed because of the merging of small pores. Another possibility is that they can be generated from the defects of blank saloplastics. During the heating stage of hot-pressing, bubbles could be trapped inside which led to this type of defect. The top surface and more cross-sectional SEM images of these films (Fig. S4) are also included in the ESI.†

#### 3.2.2. Pore closure (curing)

**Fig. 3:**
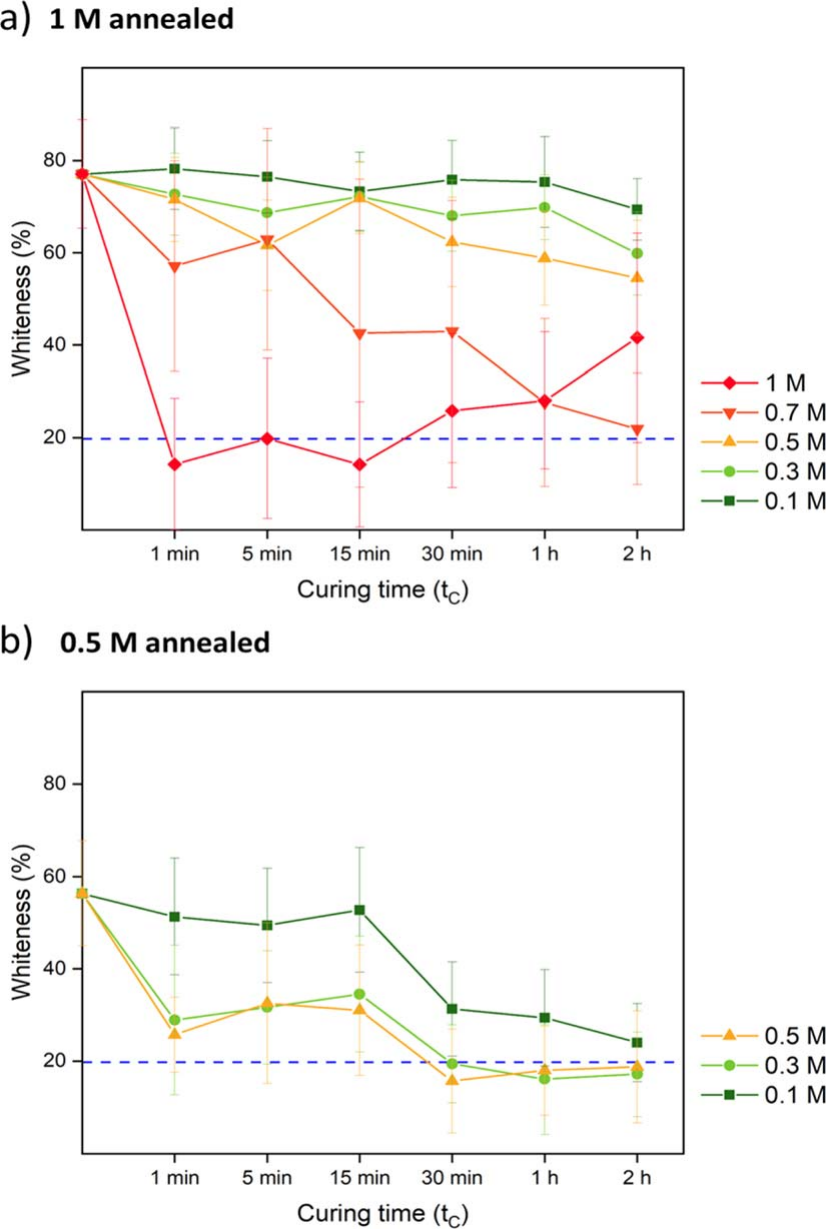
The whiteness change over time of (a) 1 M annealed and (b) 0.5 M annealed samples when cured in different concentrations of KBr. The blue dashed line represents the whiteness of the blank. The error bars represent the standard deviations of triplets.

To avoid lysozyme from escaping, the pores should be closed right after the loading step. This can be achieved by a salt curing step where chain rearrangements are induced and the crosslinking network is restored. One criterion to judge the curing step is that the final saloplastics after curing should return to the transparent state. According to the pore formation data, we chose 1 M and 0.5 M KBr annealed samples for further curing tests. As shown in Fig. 3, both samples were cured in different concentrations including and below the annealing concentration. For 1 M annealed samples, the concentration should be ≥0.7 M to be able to reduce the whiteness within 2 h. For 1 M cured samples, the thinner parts got cured first and the longer they stayed in this high KBr concentration, the more they became gel-like. As a result, the whiteness increased again after 30 min curing due to increased light scattering. For 0.5 M annealed samples, sufficient curing can be achieved by concentration ≥ 0.1 M. In both cases, the higher KBr concentration led to faster curing, which is desired since lysozyme has less time to escape.

**Fig. 4:**
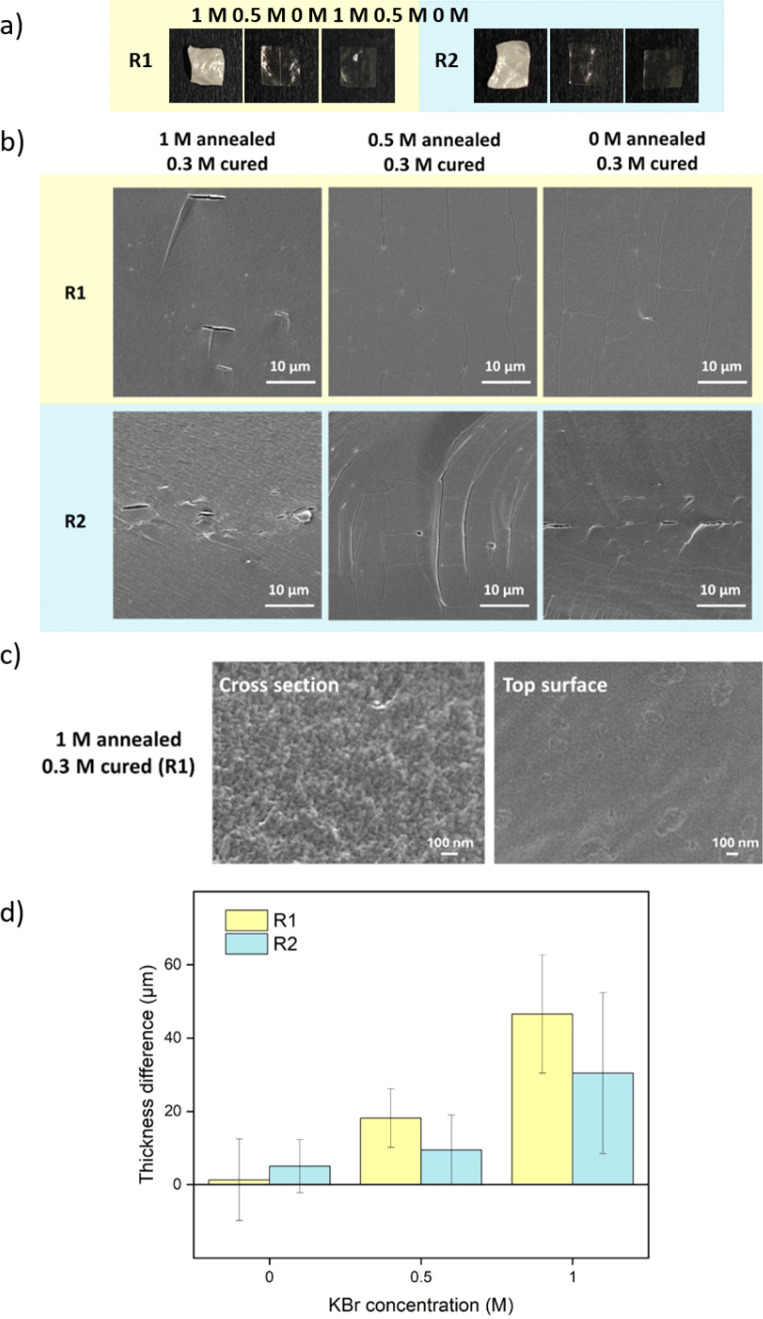
(a) Pictures and (b) cross-sectional SEM images of cured samples with different salt annealing *via* both R1 and R2. (c) Higher magnification SEM images of R1-prepared 1 M annealed then 0.3 M cured sample. (d) The thickness differences between blank dry films and cured samples. The thickness was measured from SEM cross-sectional images. The error bars represent the standard deviations of at least 5 measured points.

For comparison, samples were also prepared *via* R2 with salt annealing concentrations of 0, 0.5, and 1 M KBr. Instead of going into water, R2 samples directly got cured in a lower concentration of KBr solution. The curing concentration for both routes was set to 0.3 M, which was close to the original salt level. The annealing and curing times were both 1 h. As shown in Fig. 4a, 1 M annealed samples prepared *via* both routes remained white since 0.3 M KBr curing for 1 h was not sufficient to close the pores. For 0.5 M and 0 M annealed samples, 0.3 M KBr was enough to regain transparency. For R2, when 0.5 M annealed samples entered 0.3 M curing solution, it turned turbid then quickly returned back to transparent again. This suggests that there was still pore formation when the concentration gap was 0.2 M. When comparing Fig. 2c and 4b, most of the pores disappeared after curing except some bigger defects. For 1 M annealed sample, since it stayed in 0.3 M KBr + 20 wt% glycerol for 4 h for SEM sample preparation, the final samples returned to transparent. Besides, storing dry samples at 1 M annealing KBr concentrations might cause crystallization (Fig. S5†). As shown in Fig. 4c, 1 M annealed then 0.3 M cured sample showed a dense structure at higher magnification for both cross-section and top surface images. To further confirm the dense structure, the pure water permeabilities of blank saloplastics and R1-treated 1 M annealed then 0.3 M cured samples were measured following the literature (Fig. S6†).^69^ Both of samples showed zero water permeation at 1 bar after 5 h. As shown in Fig. 4d, the cured samples showed an increase in overall thickness and roughness compared to dry blanks, resulting in a difference in thickness between pre- and post-cured films. For 0 M annealed samples, the thickness differences induced *via* R1 or R2 were close. For higher concentrations, R1 prepared samples were more open during the water soaking step which resulted in thicker films than R2 prepared samples. Top surface and more cross-sectional SEM images of these films (Fig. S7) are also included in the ESI.†

#### 3.2.3. Determination of functionalization parameters

To perform the functionalization, we now evaluate all the parameters for each step in two different routes. According to the results, the speed of salt diffusion highly depends on the salt concentration, which is consistent with the literature.^70^ By playing with this concentration, we have demonstrated successful pore formation and closure. It is expected that this opening and closing of the pores is important for lysozyme take-up by the saloplastics. Hot-pressed saloplastics contained roughly 0.3 M KBr. For pore formation, a high KBr concentration ≥ 0.5 M was required, while for curing, the concentration should not exceed 0.3 M. Specific KBr concentrations for each step were determined as summarized in Table 1. Timewise, 1 h was sufficient for both annealing and curing, and loading lysozyme was performed overnight (∼12 h). Samples without annealing and/or curing were also prepared for comparison.

### 3.3. Effect of PDADMAC : NaPSS ratio

To investigate whether charge is important for the uptake of the positively charged lysozyme molecules, samples with extra NaPSS were also prepared to add more negative charges. As observed, when complexes were prepared at a ratio of PDADMAC : NaPSS 1 : 1, a clear supernatant could be obtained. While for other ratios, supernatants became turbid indicating the leaching of extra PECs. When preparing PDADMAC : NaPSS at a ratio of 1 : 2 in solution, the final PEC ratio was around 1 : 1.4.^71^ Thus, the differences between all ratios were limited.

**Fig. 5:**
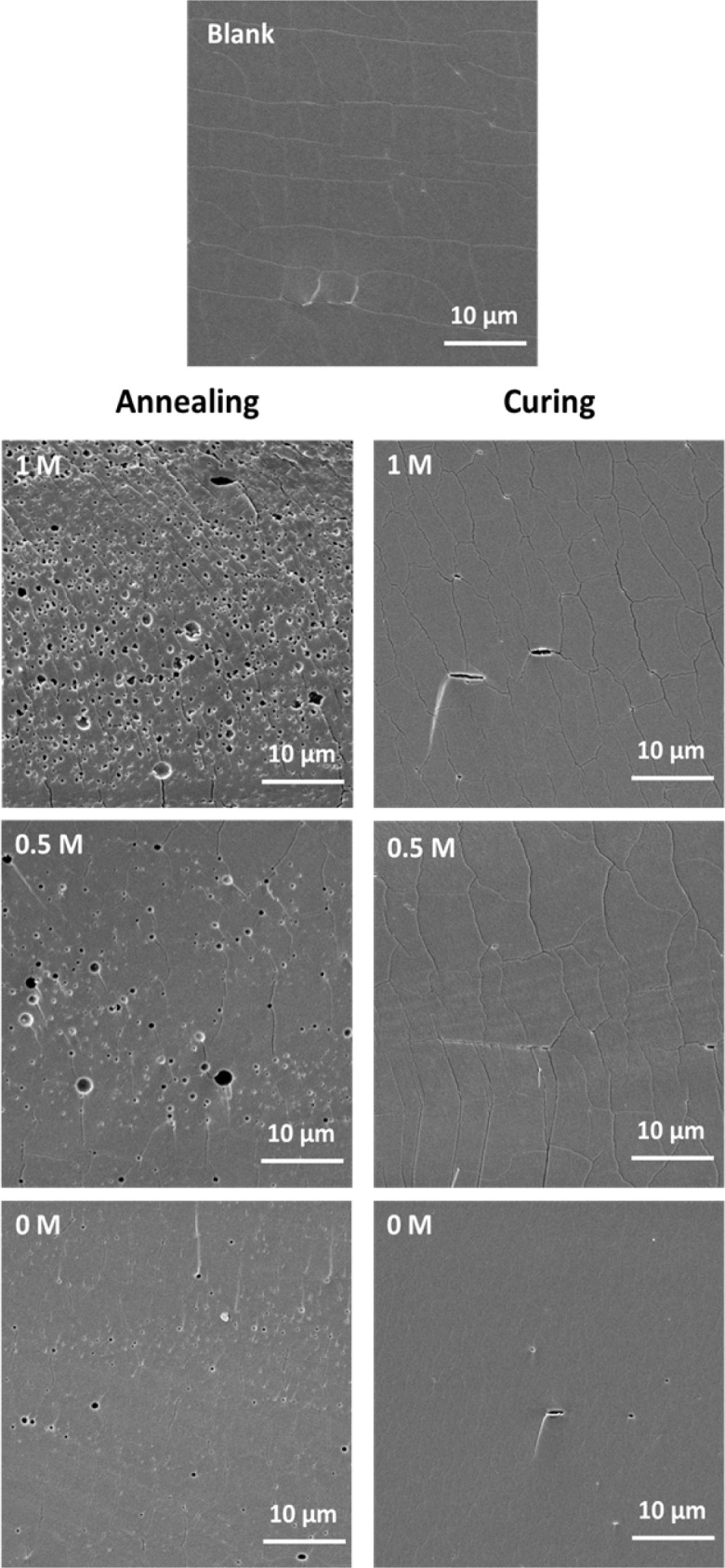
Cross-sectional SEM images of samples at a PDADMAC : NaPSS ratio of 1 : 2.5 after annealing and curing.

We have studied the salt treatments for PDADMAC : NaPSS at a ratio of 1 : 1 *via* R1. For other PDADMAC : NaPSS ratios 1 : 1.5, 1 : 2, and 1 : 2.5, there should be a slight difference in whiteness and required treatment time. For comparison, the same annealing and curing were performed for all ratios. Here, we show the SEM images of 1 : 2.5 samples as an example (Fig. 5) that pores can be formed then cured. There was no significant difference when comparing different ratios. The images of other ratios were summarized in Fig. S8.† All ratios could be further studied for lysozyme loading.

### 3.4. Determination of lysozyme concentration for loading

Next crucial parameter is to determine a suitable lysozyme concentration for sufficient loading. To get a detectable difference in absorbance, 10 pieces of 1 cm^2^ saloplastics at a ratio of 1 : 1 were soaked in 20 mL lysozyme solutions. These saloplastics were first annealed in 1 M KBr since the most porous structure should have the maximum loading. Different concentrations of lysozyme were studied and as shown in Fig. S9,† the loading at 50 mg L^−1^ started to show a detectable absorbance loss. The provided lysozyme for each 1 cm^2^ piece at this concentration and volume was 0.1 mg per piece. According to the absorbance loss of 50 mg per L solution and the extinction coefficient of lysozyme (Fig. S10†), around 2.7 μg lysozyme was actually loaded per piece (eqn (S3)†). During the preparation, it was observed that 1 mL solution was sufficient to soak one piece. Since shifting to different ratios, which contained more NaPSS may increase the loading, the concentration was increased to 500 mg L^−1^ to ensure sufficient loading and the volume was reduced to 10 mL for 10 pieces. The calculated available lysozyme was then 0.5 mg per piece. After finding the best ratio and salt treatment, lower concentrations of lysozyme were also tested.

### 3.5. Lysozyme activity

Before examining the enzymatic activity of lysozyme, some supplementary experiments were performed. The presence of free PEs, both PDADMAC and NaPSS, had a negative impact on the lysozyme activity (Fig. S11†), which is consistent with the literature.^44^ On the contrary, the presence of KBr seems to have no effect on the enzymatic activity. This was further tested by storing lysozyme in different concentrations of KBr solutions for up to 7 days and no significant decrease in the enzymatic activity was observed (Fig. S12†). Lysozyme activity could be inhibited while stored in a high KBr (1 M) concentration solution but this concentration was not strong enough to fully denature lysozyme.^72–74^ We therefore expect the KBr concentrations used in R2 will not affect the enzymatic activity of lysozyme. We also measured the influence of the KBr concentration on the *Micrococcus lysodeikticus* assay (Fig. S13†) and no significant effect was observed.

#### 3.5.1. Route 1 *vs.* route 2

To examine the enzymatic activity of lysozyme, *Micrococcus lysodeikticus* assay was used. Lysozyme breaks down the bacteria which leads to a decrease in the absorbance at 450 nm. All blank saloplastics at different PDADMAC : NaPSS ratios were measured first and they showed only limited adsorption that the absorbance at 450 nm did not change much within 5 h (Fig. S14†). Furthermore, to rule out the absorption of lysozyme by swelling only, samples were annealed and loaded with 500 mg per L lysozyme in 0.3 M KBr, then cured and washed with 0.3 M KBr (AL0.3 C0.3). The salt concentration was kept constant thus no pore formation was observed, and the samples remained transparent. They only showed limited activity against the bacteria (Fig. S15†).

**Fig. 6:**
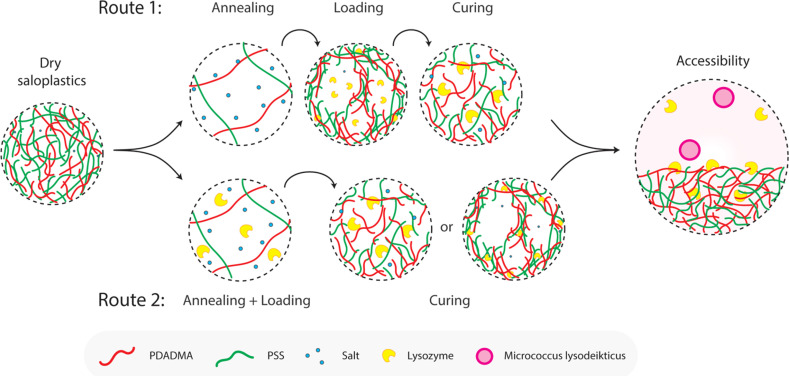
Proposed mechanisms for R1 (static incorporation) and R2 (dynamic incorporation). The accessibility between lysozyme and substrate also plays a vital role in determining the enzymatic activity.

Two different routes as shown in Fig. 1 were proposed to capture lysozyme and the possible mechanisms are discussed here in Fig. 6. To explain the influence of annealing and curing, Fig. 6 demonstrates the possible mechanisms for capturing lysozyme *via* route 1 (R1) or route 2 (R2). For R1, pores were created and then became available to incorporate lysozyme. This process is a more static incorporation where strong attraction between lysozyme and saloplastics is preferred. Higher annealing concentrations would lead to more and larger pores, which is beneficial for loading more lysozyme. However, if not properly cured, most of lysozyme would leak during the washing stage before the measurement. In the case of R1, lysozyme can be located both inside the pores and the walls. For R2, lysozyme was pre-filled into the pores of the saloplastics and during the curing step, the lysozyme could be trapped immediately inside the shrunk pores or walls according to the concentration gap. This process is more dynamic therefore lysozyme should have less time to escape and distribute more homogenously within the saloplastics. The same curing times in 0.3 KBr solution were used for both R1 and R2 samples. For a lower KBr concentration, faster curing is expected, preventing lysozyme leakage. The enzymatic activity not only depends on the amount of lysozyme but also its accessibility to the *Micrococcus lysodeikticus* substrate, which has a diameter around 0.5–2 μm.^75^ During the measurement, lysozyme could be released from the saloplastics which would have the highest accessibility with the substrate indicating a higher activity compared to lysozyme located on the surface or within the saloplastics.

**Fig. 7:**
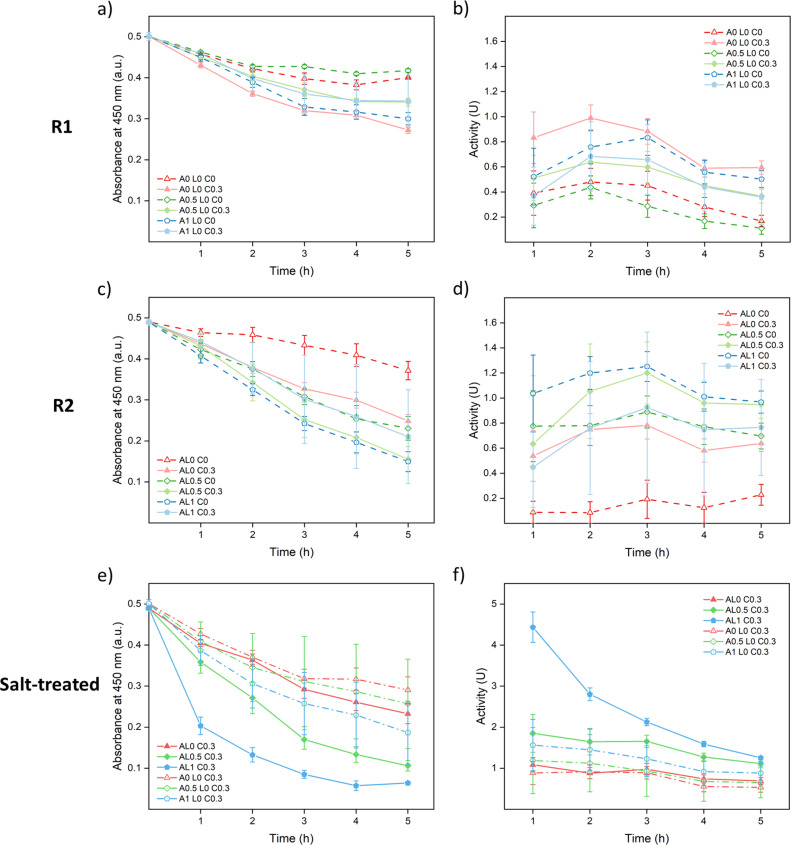
Absorbance change and enzymatic activity of lysozyme functionalized saloplastics at a PDADMAC : NaPSS ratio of 1 : 2.5: (a) and (b) are samples prepared *via* R1; (c) and (d) are samples prepared *via* R2; and (e) and (f) are R1 and R2 samples pre-treated with 150 μL of 1 M KBr. The error bars stand for the standard deviations of three samples.

As shown in Fig. 7a and b, A0 L0 C0.3 samples showed the best enzymatic activity among R1 samples since they were the best cured. The cured samples showed better performances than the uncured samples except A1 L0 C0.3. A possible reason is that since 0.3 M was not enough to fully cure the 1 M annealed samples as shown in Fig. 3a, part of the lysozyme was washed away just as A1 L0 C0 samples. Meanwhile, curing could close up the structure causing hindrance between lysozyme and the substrate which lowered the accessibility. As shown in Fig. 7c and d, similar trends were observed since curing helped to prevent leakage except for AL1 C0.3. Among R2 samples, AL1 C0 showed the best performance since it captured more lysozyme and had a more open structure for accessibility.

For both R1 and R2 samples, the highest activity was achieved around 2^nd^ or 3^rd^ hour during the measurements (Fig. 7b and d) which suggests that the saloplastics took time for swelling to get in contact with the bacteria. It was noticed that for all 0.3 M KBr cured samples, the saloplastics were only slightly white during measurements. This was caused by the small salt gap from 0.3 M KBr to 50 mM PBS solution. To open up the structure and increase the accessibility, one method was to increase the temperature. However, as shown in Fig. S16,† the blank saloplastics were not stable at 45 °C and leaked PECs/PEs which showed a coagulation effect on the bacteria. Thus, instead of increasing the temperature, 150 μL of 1 M KBr solution was added to each cured piece and soaked for 5 min before the enzymatic activity measurement. With this salt treatment, the accessibility between lysozyme and substrate was maximized which helped to show how much lysozyme was actually loaded. All cured samples appeared white and showed improved activity as shown in Fig. 7e and f. Since the structure was already open, the highest activity was achieved at the 1^st^ hour. Overall, samples prepared *via* R2 contained more lysozyme than R1. This indicates that dynamic incorporation works more efficiently than the static incorporation since the lack of attraction between lysozyme and saloplastics. Among all cured samples, AL1 C0.3 showed the best activity since it captured the most lysozyme.

#### 3.5.2. Activity *vs.* different polyelectrolyte ratios

**Fig. 8:**
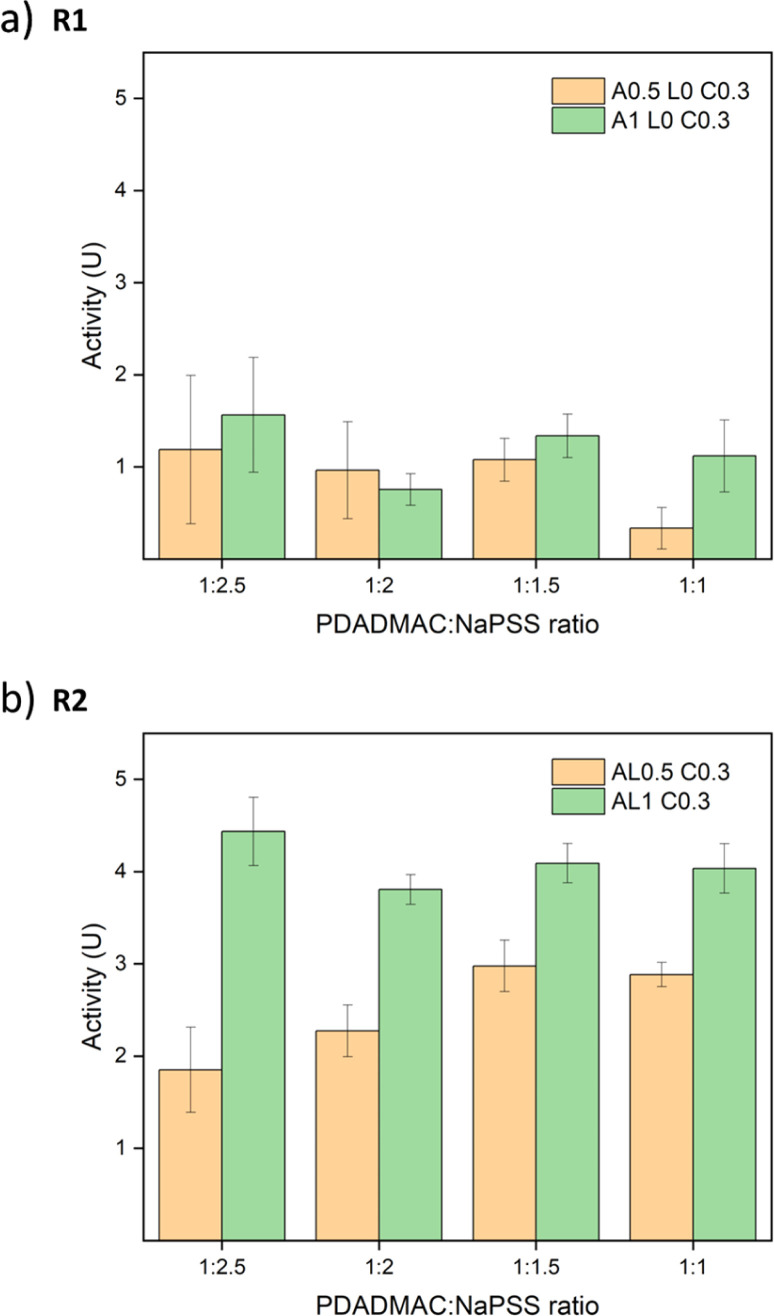
Enzymatic activity of salt-treated samples *vs.* different PDADMAC : NaPSS ratios. The error bars represent the standard deviations of three samples.

The same activity assays were performed for other PDADMAC : NaPSS ratios of 1 : 2, 1 : 1.5, and 1 : 1. All absorbance graphs of these other ratios are provided in Fig. S17.† Similar trends are observed and all R2 samples showed better performance than R1 samples. To compare the different ratios, the best activities of salt-treated samples are summarized in Fig. 8. For R1 samples, the differences between 0.5 M annealed and 1 M annealed samples were small except samples at a ratio of 1 : 1 since the overall activity was low and showed large errors. For R2 samples, 1 M annealed samples all showed better activity than 0.5 M annealed samples. Contradictory to our assumption, the ratio effect was not significant. Although the addition of PSS may increase the net negative charge of the complex, the stability of the saloplastics was weakened. It could be that during the extensive salt annealing and curing steps, the extra PSS chains were released bringing the saloplastics back to a net neutral charge. Among all samples, the best activity was 4.44 ± 0.37 U cm^−2^, which is comparable to other studies.^44,76,77^

#### 3.5.3. Activity *vs.* lysozyme loading concentration

**Fig. 9:**
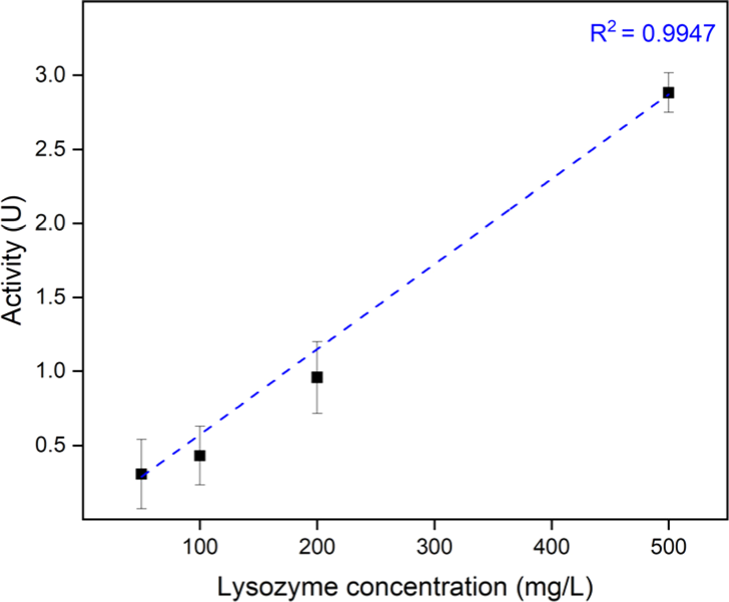
Enzymatic activity of AL0.5 C0.3 samples *vs.* loading lysozyme concentration. The error bars represent the standard deviations of three samples.

According to all activity results, R2 showed more effective capturing of the lysozyme than R1. The differences among different ratios were not significant, while preparing saloplastics at a PDADMAC : NaPSS ratio of 1 : 1 would waste the least NaPSS during the complexation. AL1 C0.3 showed the best activity, however, with much higher roughness and whiteness since it was not fully cured. Thus, AL0.5 C0.3 samples at a PDADMAC : NaPSS ratio of 1 : 1 *via* R2 were used to further study the effect of lysozyme loading concentration. To open up the saloplastics, same salt treatment with 150 μL 1 M KBr was performed. As shown in Fig. 9, a linear relationship was found between the loading lysozyme concentration and the final enzymatic activity. It is possible to increase the activity even higher by increasing the loading concentration.

### 3.6. Lysozyme stability

**Fig. 10:**
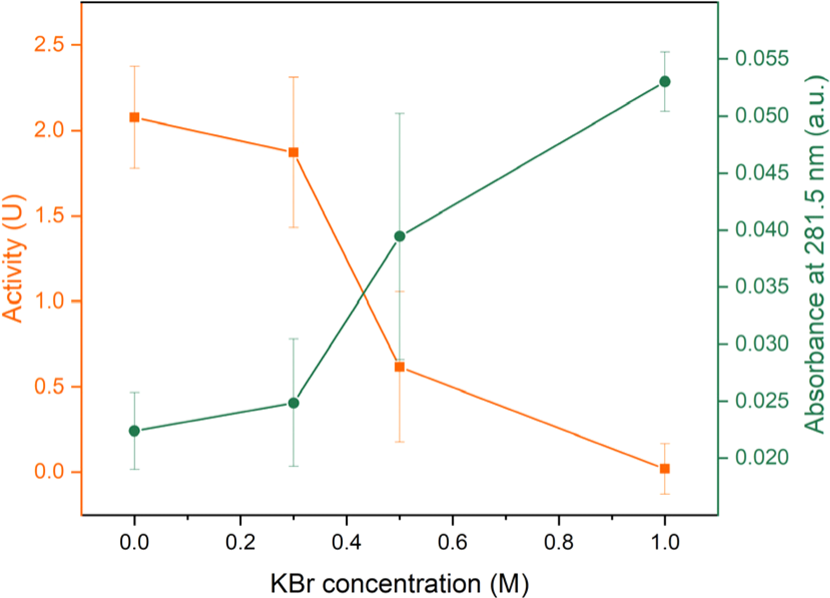
Enzymatic activity of AL0.5 C0.3 samples at day 7 after storing in water and different concentrations of KBr solutions. The absorbance at 281.5 nm of the supernatants were also measured. The error bars represent the standard deviations of three samples.

Considering all aspects mentioned in Section 3.5, AL0.5 C0.3 samples at a PDADMAC : NaPSS ratio of 1 : 1 *via* R2 were used to study the stability over time and over different KBr concentration. The loading lysozyme concentration was 500 mg L^−1^. To induce a salt gap, 150 μL 1 M salt was again used for opening up saloplastics. For samples stored at dry conditions, the average activity of these samples was 1.96 ± 0.22 U cm^−2^, which showed around 68% preservation of the activity compared to day 0. When stored in water, around 72% of the activity was preserved as shown in Fig. 10. However, with the addition of KBr, the activity kept reducing suggesting the release of lysozyme into the solutions. At 1 M KBr, almost no activity was detected. This outcome was expected since lysozyme was not covalently bound to PECs. At high salt concentrations, PECs regained flexibility and lysozyme was released driven by a concentration difference. This result was consistent with the detected amount of lysozyme in the supernatant. As shown in Fig. 10, the absorbance at 281.5 nm kept increasing when increasing the KBr concentration. At 1 M KBr, the assumption was that all lysozyme in the saloplastics was released. According to the absorbance of supernatant and the extinction coefficient of lysozyme, it can be estimated that each 1 cm^2^ piece contained around 26 μg lysozyme (Fig. S10†). This estimation is also consistent with Section 3.4, where the loading concentration was 50 mg L^−1^ and the loaded lysozyme was ∼2.7 μg.

To sum up, the lysozyme-functionalized saloplastics show enzymatic activity. Here, observed differences in enzymatic activities result from both the different structure of saloplastics and different amount of incorporated lysozyme. The precise quantification of lysozyme within the structure and its distribution is difficult. Hedberg *et al.* pointed this out that only few papers reported the precise amount of immobilized proteins.^78^ For the detection of lysozyme, there are many characterization methods, such as bicinchoninic acid assay,^77^ enzyme-linked immunosorbent assay,^79^ western blotting,^80^ HPLC,^81^ and fluorescence microscopy.^82^ The difficulty is that there should be sufficient amount of lysozyme to reach the limitation of the detection or labels have to be used that could affect the enzymatic activity and lysozyme uptake. Also, for some characterizations, lysozyme should be separated from the matrix since the presence of polyelectrolytes could interfere with these measurements. For morphological characterizations, other techniques could be used, such as (cryo)TEM and AFM.^83^ We recently developed a label free NMR based method that can be used to measure the complete mass balance of PECs.^84^ In the near future, we are planning to use this method to determine the composition of saloplastics and polyelectrolyte complexes with and without proteins to obtain further fundamental understanding of protein uptake by polyelectrolyte complexes. It is also important to characterize the release of lysozyme under various conditions to study the long-term stability and life time of these functionalized saloplastics.

## 4 Conclusions

In summary, we have demonstrated a simple but effective way to incorporate lysozyme into hot-pressed saloplastics. From the whiteness and SEM data, successful pore formation and closure were achieved by manipulating the salt concentration gap. Two routes of lysozyme loading were systematically examined and the dynamic capturing method showed overall better enzymatic activities. The saloplastics at a PDADMAC : NaPSS ratio of 1 : 2.5 has shown the highest activity around 4.44 U cm^−2^. After 7 days, around 72% of the activity was still preserved when stored in water. This study provides new possibilities for sustainable saloplastics and a straightforward method to functionalize them. Focusing on the potential antimicrobial properties, surface immobilization can be studied in the future to maximize the contact with microbes. Covalent crosslinking can also be performed to reduce leakage. Other inorganic components with antimicrobial properties can also be considered, such as silver nanoparticles, since they can be hot-pressed. Another important direction is to study the bio-based polyelectrolytes and the salt effects on these complexes since together with enzymes, fully biocompatible/biodegradable systems could be built.^85,86^

## Data availability

The dataset of this article are available in 4TU.ResearchData at: https://doi.org/10.4121/3eed5d20-d65e-4348-81d5-e55c79f6a064.

## Author contributions

Jiaying Li: conceptualization, data curation, investigation, methodology, writing – original draft. Lijie Li: data curation, investigation, methodology, writing – review & editing. Saskia Lindhoud: conceptualization, project administration, supervision, writing – review & editing, funding acquisition.

## Conflicts of interest

There are no conflicts to declare.

## Supplementary Material

RA-014-D4RA04986A-s001

RA-014-D4RA04986A-s002

RA-014-D4RA04986A-s003
