# Supplementary material for: Achieving lysozyme functionalization in PDADMAC–NaPSS saloplastics through salt annealing
Source: RSC Adv. 2024 Oct 18;14(45):32863–75. doi: 10.1039/d4ra04986a (PMC11487472; doi:10.1039/d4ra04986a)
Supplement: RA-014-D4RA04986A-s003 [file RA-014-D4RA04986A-s003.pdf]

Supplementary Information (SI) for RSC Advances.
This journal is © The Royal Society of Chemistry 2024

Electronic Supplementary Material (ESI) for RSC Advances.
This journal is © The Royal Society of Chemistry 2024

SUPPORTING INFORMATION

Achieving Lysozyme Functionalization in PDADMAC-NaPSS Saloplastics through Salt Annealing

*Jiaying Li, Lijie Li, Saskia Lindhoud**

1. Department of Molecules and Materials, MESA+ Institute for Nanotechnology, University of Twente, Faculty of Science and Technology, P.O. Box 217, 7500 AE Enschede, The Netherlands

** Corresponding Author Saskia Lindhoud Email: s.lindhoud@utwente.nl*

Whiteness extraction method

After salt annealing and soaking in water, pictures of samples were taken using a black background. Surface water was gently removed and pictures were taken quickly to avoid pore collapsing. The same black background was used and all the pictures were taken at the same spot with the same lighting condition. Then, the pictures were processed using ImageJ software to extract whiteness information following Figure S1.

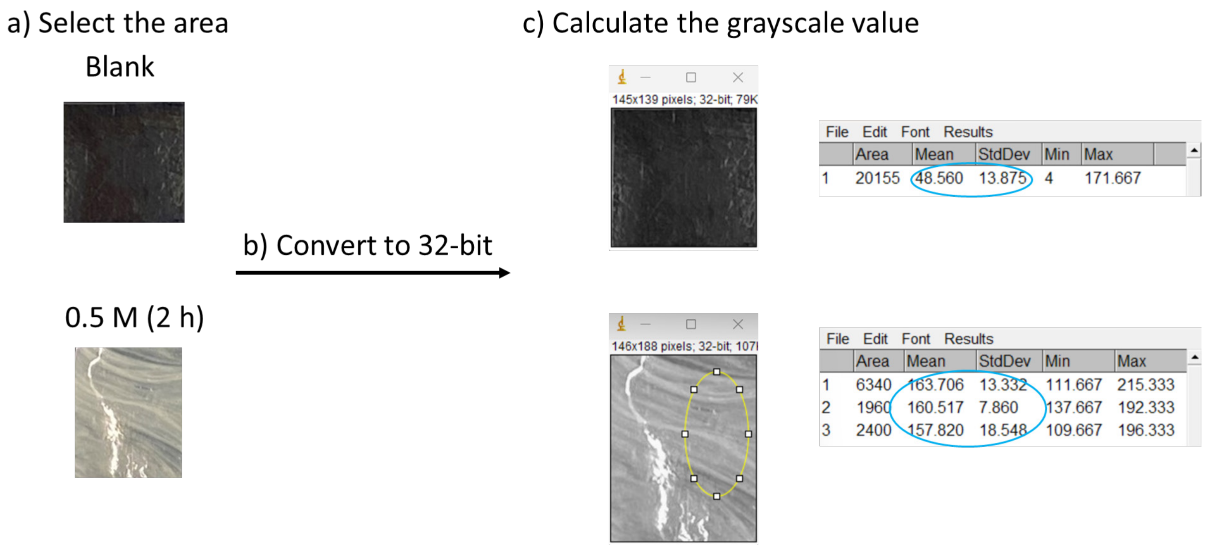

Figure S1. Whiteness extraction using ImageJ: a) Pictures were cropped to the areas that need to be detected. Here, one blank sample (top) and one 0.5 M-2 h-annealed (bottom) sample are used as examples. b) The original pictures were processed to black and white 32-bit images (Image → Type → 32-bit). c) The grayscale value of the whole selected area can be calculated using Analyze → Measure. Homogeneous areas of the film were selected, bright light-refracting areas as shown in the 0.5 M annealed sample, indicating surface roughness, were not processed.

As shown in Figure S1, grayscale values can be extracted. Then for each data point, 3 samples were measured. In the RGB system, pure black is (0,0,0), while pure white is (255,255,255). The degree of whiteness (%) of films can be calculated based on Equation S1:

$$Whiteness\ (\%) = \frac{Obtained\ value}{255} * 100 \tag{S1}$$

The black background used was not purely black which had a whiteness ~ 8%.

Preparation of saloplastics

The saloplastics were prepared following Krishna B *et al.*'s method.

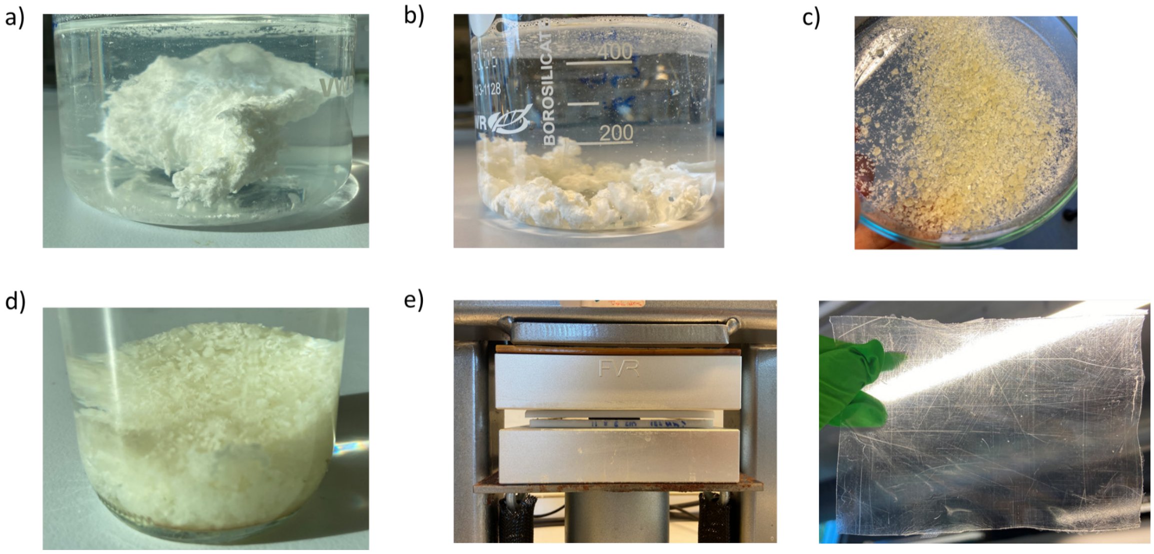

Figure S2. The preparation of PDADMAC-NaPSS saloplastics: a) Bulk complex was formed by directly mixing two polyelectrolyte solutions. Here, PDADMAC:NaPSS at a mixing monomer ratio of 1:1 is shown as an example. b) Bulk complex was broken into smaller pieces for washing. c) Complex was dried and further processed into small granules. d) Upon hot-pressing, solid complex was swelled in a 0.5 M KBr solution. e) After hot-pressing, a dense transparent saloplastic film was obtained.

Water uptake of prepared saloplastics

The water uptake of dry saloplastics was measured by soaking dry pieces in water for 24 h. The water uptake (%) was calculated by Equation S2:

$$Water\ uptake\ (\%) = \frac{m_{wet} - m_{dry}}{m_{dry}} * 100 \qquad \text{(S2)}$$

where m_{dry} is the weight of samples dried in the oven at 80 °C for 4 h and m_{wet} is the weight of wet samples. Extra surface water was carefully removed by tissues. 3 samples from different films were measured and the average results are reported with standard deviations.

Whiteness change when immersed in 0.1 M vs 0.3 M KBr

a) 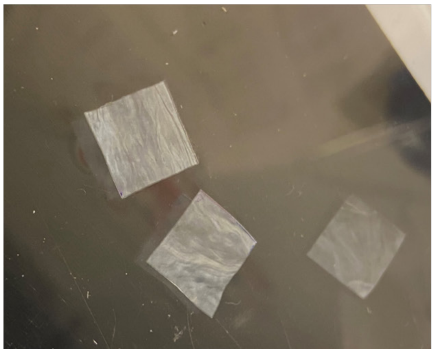 b) 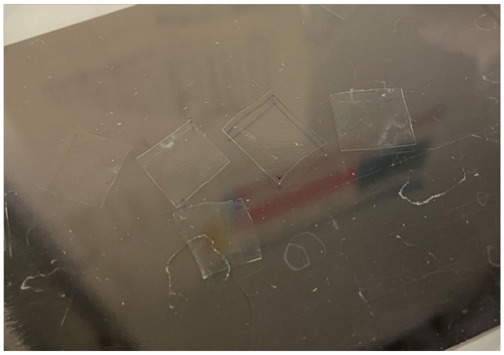

Figure S3. When immersed in 0.1 M KBr, the saloplastics first turned white indicating the release of salt (a), which suggesting that the original salt concentration was higher than 0.1 M. This change was not observed for saloplastics immersed in 0.3 M KBr, indicating that the original salt concentration was ≤ 0.3 M (b).

SEM of salt-annealed samples after soaking in water

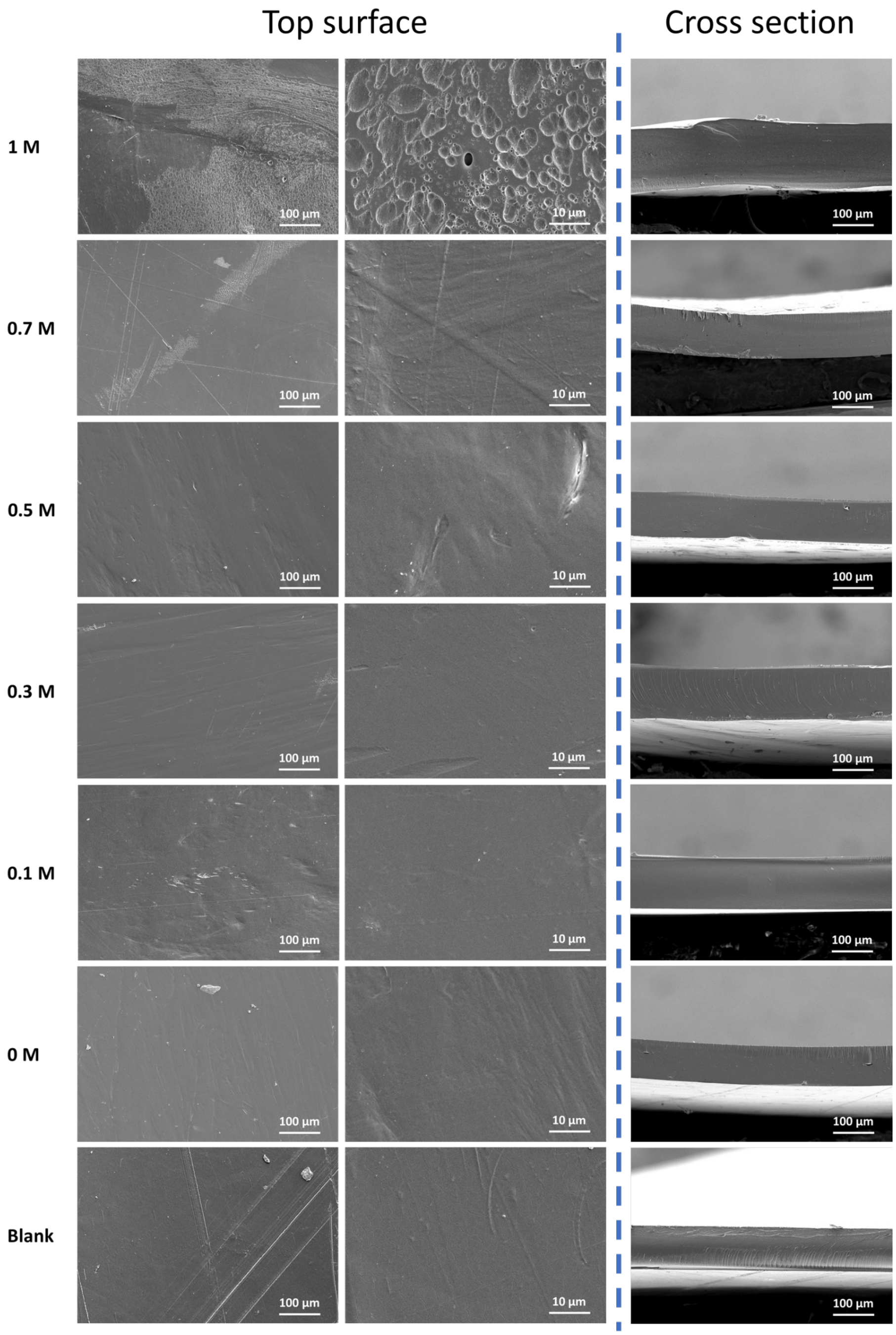

Figure S4. SEM images (left two images: top surface, right image: cross-section) of saloplastics annealed in different salt concentrations, followed by soaking in water. From the top surface images, there were no visible pores except for 1 M samples. The smaller pores could have collapsed during drying.

Dry storage at higher concentrations of salt

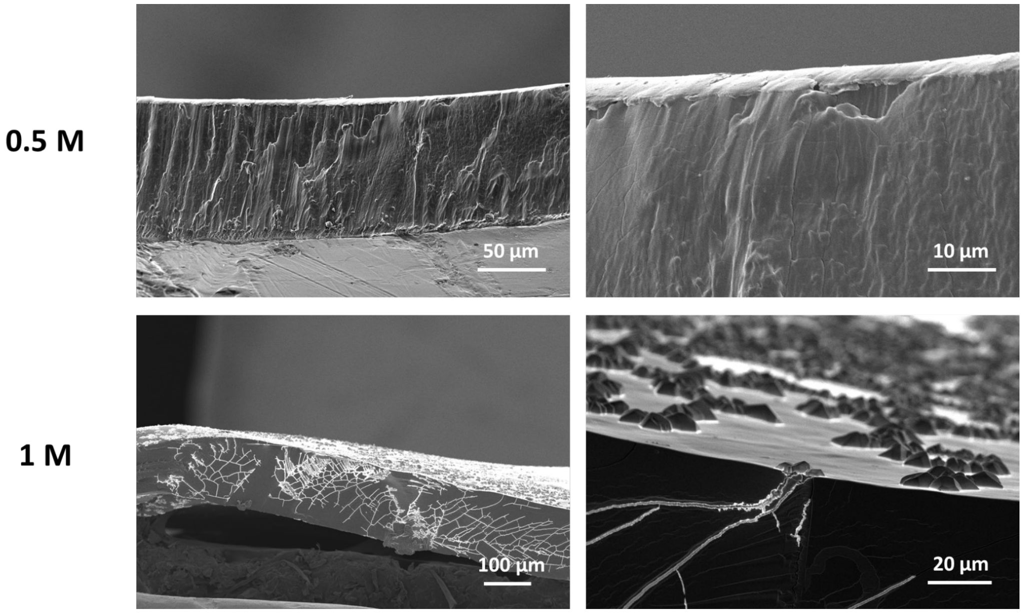

Figure S5. Cross-sectional SEM images of salt-annealed saloplastics at a ratio of 1:1. After drying overnight, 0.5 M KBr annealed samples appeared normal, while 1 M KBr annealed samples showed salt crystallization on the surface and across the film.

Pure water permeability tests

The pure water permeability tests were conducted following the literature. As shown in Figure S6, both blank saloplastics and cured samples (1 M annealed 0.3 M cured via R1) were cut into ~ 2.5 cm circles. For better flexibility, blank saloplastics were rinsed with water right before cutting. A non-woven piece was used as a support. The samples were mounted on a dead-end filtration cell and the feed water was pressured at 1 bar by nitrogen. If there would be any permeated water, this would be collected, for the saloplastics, there was no permeation for 5 h, indicating a dense and non-porous structure.

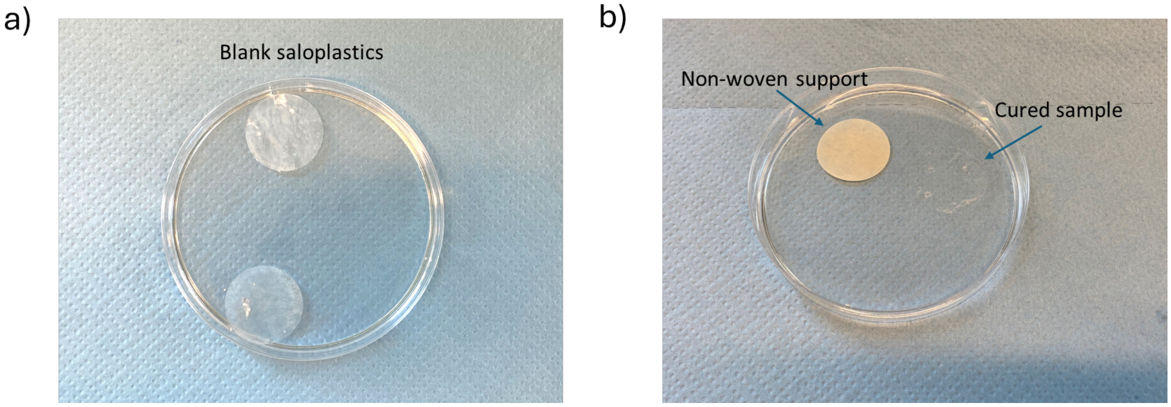

Figure S6. Pictures of pure water permeability samples a) blank saloplastics and b) cured sample (1 M annealed 0.3 M cured via R1). A non-woven porous piece was used in all measurements to provide support.

SEM of salt-cured samples

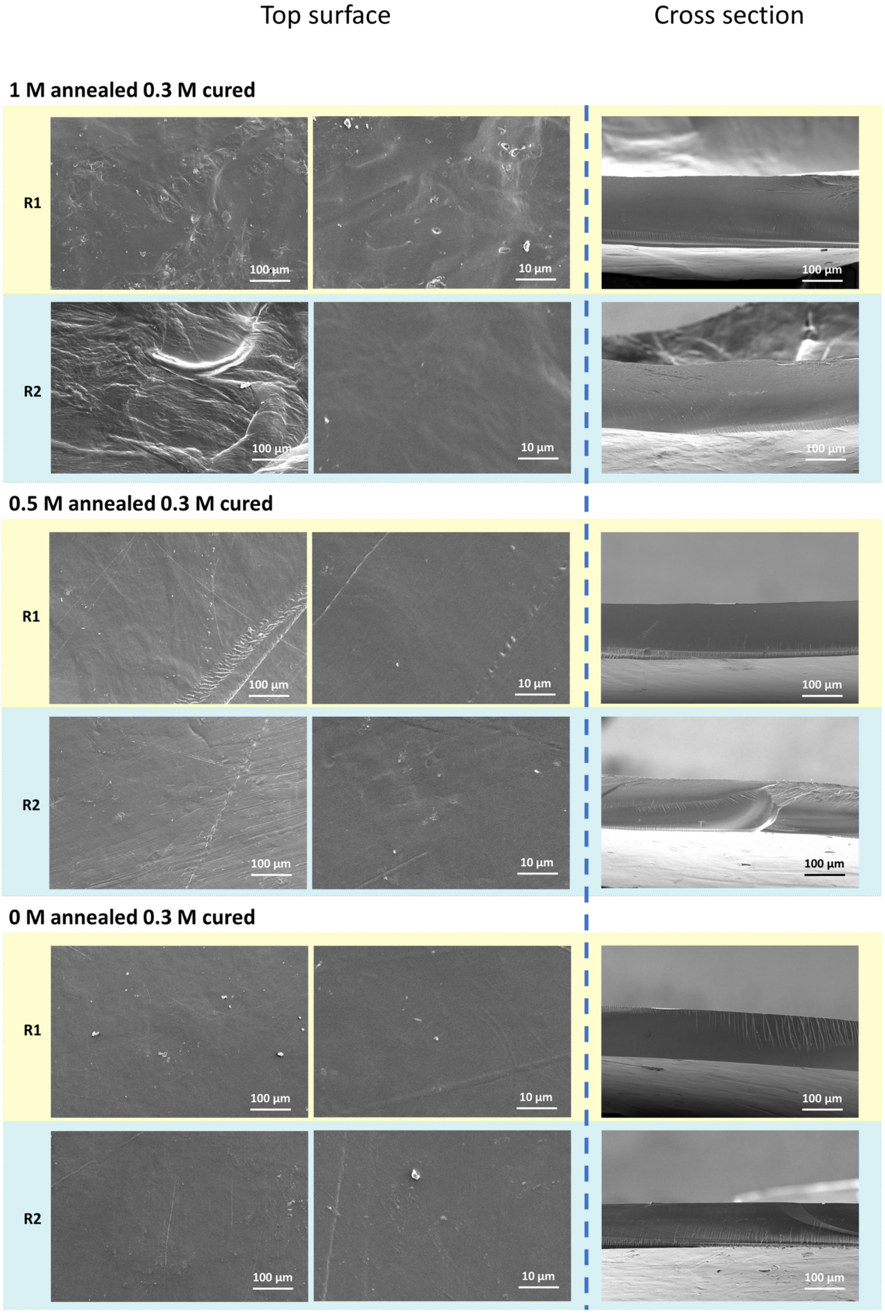

Figure S7. SEM images (left two images: top surface, right image: cross-section) of different salt concentration annealed saloplastics cured in 0.3 M KBr. These cross-sectional images were used to measure the thickness of samples. After SEM sample preparation, the thickness might be thinner than real thickness since the samples were under vacuum.

SEM of samples at other PDADMAC:NaPSS ratios

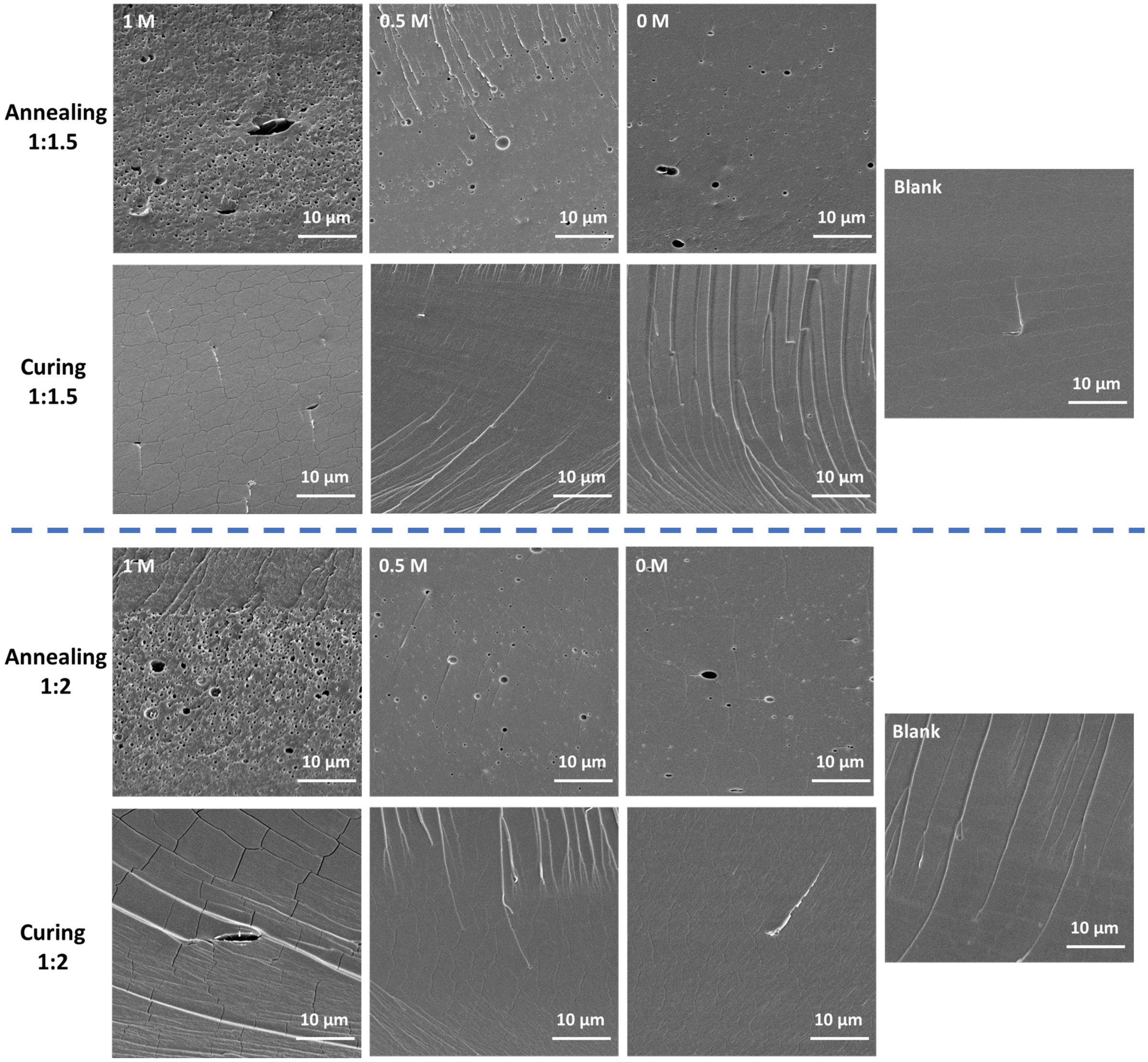

Figure S8. Cross-sectional SEM images of annealed and cured saloplastics at PDADMAC:NaPSS ratios of 1:1.5 and 1:2. They both showed successful pore formation and closure.

Absorbance loss at 281.5 nm after loading

By comparing the absorbance loss in the lysozyme solutions before and after loading saloplastics, the amount of loading can be estimated. When using 5 and 10 mg/L, there were increases in the absorbance. The possible reason was that PECs could leak into the solution which also gave a signal around 281.5 nm. When soaking these saloplastics in only water, there was an absorbance of 0.0184 ± 0.005. Another reason is due to the equipment resolution limit that it was less accurate at extremely low concentrations. To get a detectable value in absorbance, the loading concentration should be at least 50 mg/L.

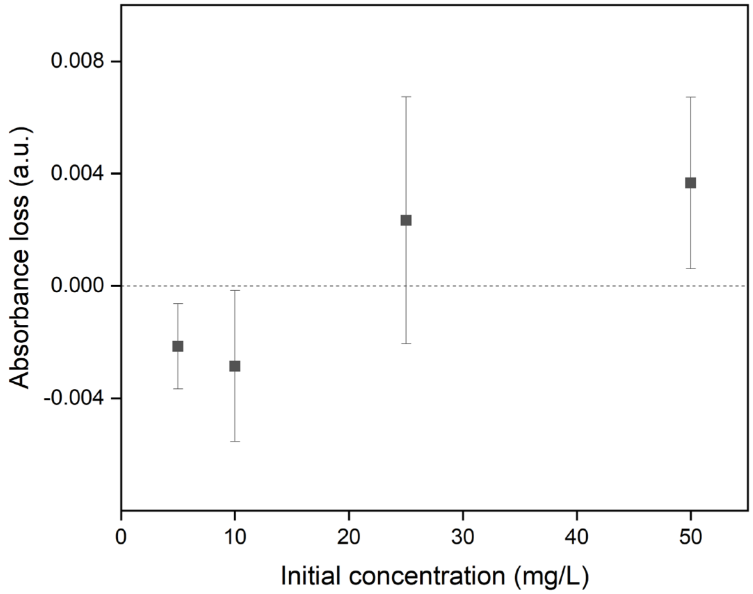

Figure S9. Different concentrations of lysozyme solutions were used for loading saloplastics. An absorbance loss was calculated to estimate how much lysozyme was loaded.

Extinction coefficient of lysozyme solutions

The extinction coefficient (L/g·cm) of lysozyme is 2.635. From our calculation based on Figure S10, the value was slightly different (2.7, $R^2 = 0.9988$) and it was stable after 7 days. For one piece of 1 cm^2 functionalized saloplastics, the contained lysozyme was around 2.7 ± 2.3 µg with 50 mg/L lysozyme loading solution, according to Figure S9.

Calculation:

$$c = \frac{\Delta A}{\varepsilon L} \tag{S3}$$

where ΔA is the absorbance loss at 281.5 nm before/after loading, ε is the extinction coefficient (2.7 L/g·cm from Figure S10), and L is 1 cm.

ΔA with 50 mg/L lysozyme loading solution was 0.0037 ± 0.0031. The calculated concentration (c) according to the Equation S3 was 1.36 ± 1.13 mg/L. The volume was 20 mL. Thus, the total loaded for 10 pieces was 0.027 ± 0.023 mg. The loaded lysozyme was 2.7 ± 2.3 µg for each piece.

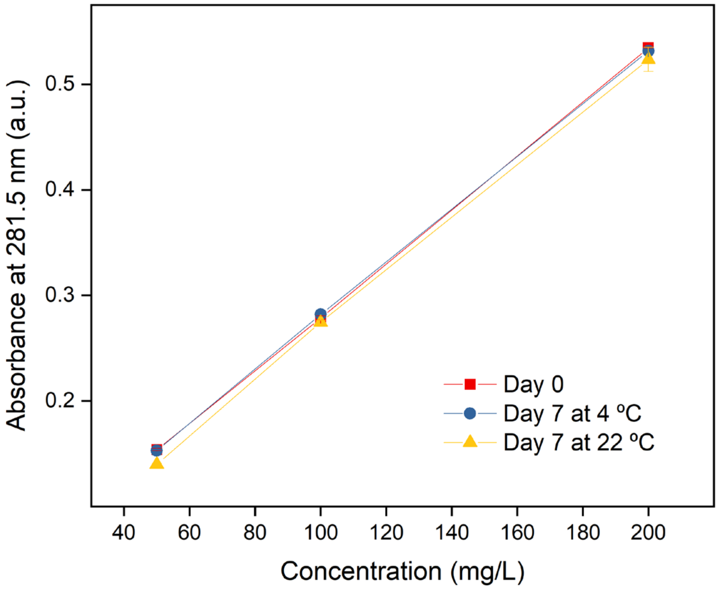

Figure S10. The absorbance at 281.5 nm of freshly made lysozyme solutions and one-week old solutions stored at room temperature and in the fridge (4 °C). There was no significant different in absorbance.

The activity of lysozyme in the presence of PDADMAC, NaPSS, or KBr

In the presence of polyelectrolytes, the activity of lysozyme both dropped to around 60% (for NaPSS 57.2 ± 3.6%, for PDADMAC 66.5 ± 9.8%). With the presence of KBr, the influence was less significant (96.7 ± 3.7%).

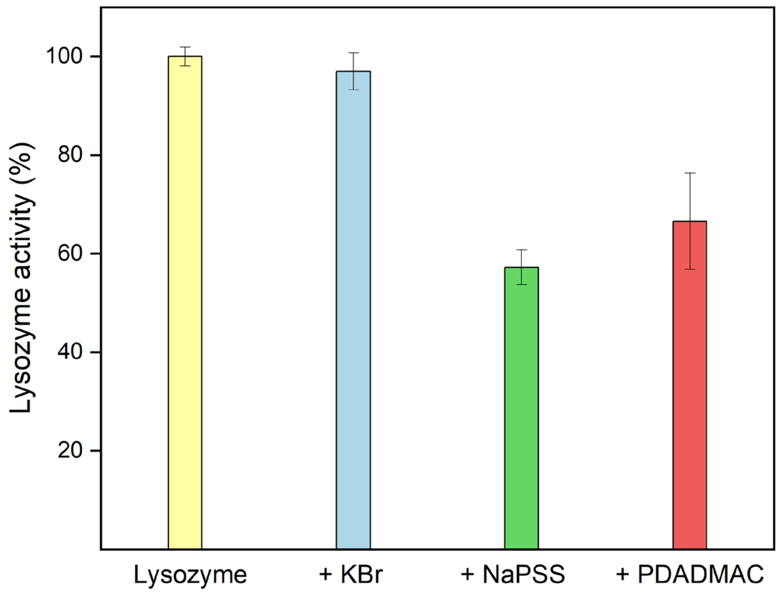

Figure S11. The influence of PDADMAC (0.1 wt%), NaPSS (0.1 wt%), or KBr (1 M) on the activity of lysozyme (10 mg/L) was examined. Activity was determined by adding 50 μL lysozyme solution with/without addition of polyelectrolytes/KBr into 2 mL of bacteria suspension. The blank was prepared by adding 50 μL PBS solution into 2 mL substrate. The experiments were conducted right after the solution preparation (Day 0). 3 samples were tested and the average results with standard deviations are reported.

Lysozyme stability vs KBr and time

Lysozyme solutions (10 mg/L) were stored in different concentrations of KBr for overnight or 7 days at 4 °C. Then, 50 μL of these solutions were added into 2 mL substrate suspension to check the activity. Salt showed no significant influence on changing the activity of free lysozyme. The lysozyme activity also showed no difference when compared to the activity of Day 0 lysozyme solution.

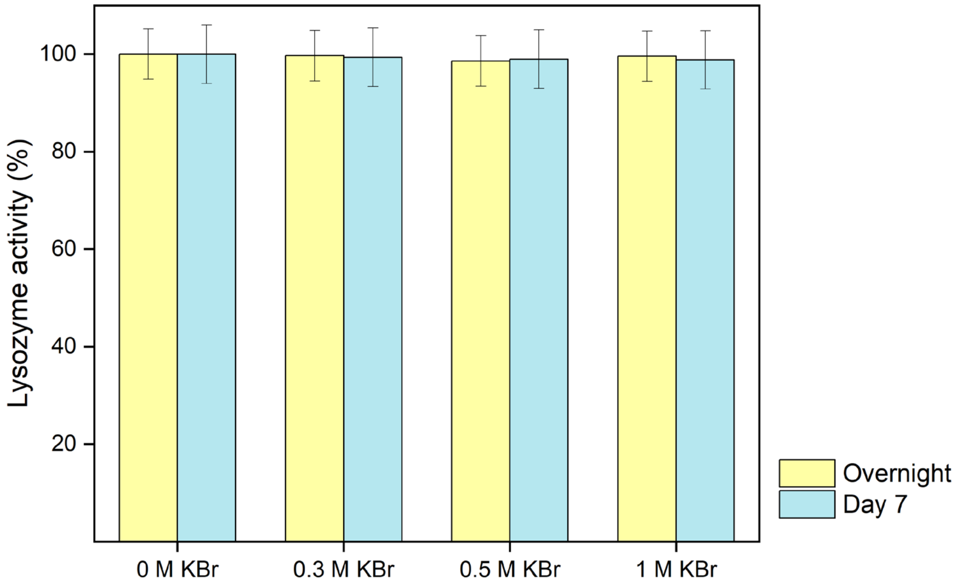

Figure S12. The influence of KBr and its concentration on lysozyme activity over time was examined.

Addition of KBr to substrate

The addition of different concentrations of KBr into the substrate suspension would not significantly affect the absorbance. A slight dilution was observed for all concentrations.

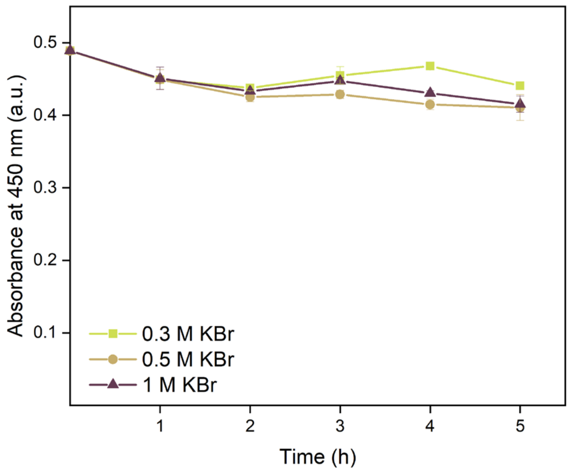

Figure S13. The influence of just KBr on the substrate was examined. The experiments were conducted by adding 50 µL KBr (0.3, 0.5, or 1 M KBr) into 2 mL substrate.

Micrococcus lysodeikticus assay of blanks

Blanks at different ratios of PDADMAC:NaPSS were also tested where a slight adsorption was observed over time.

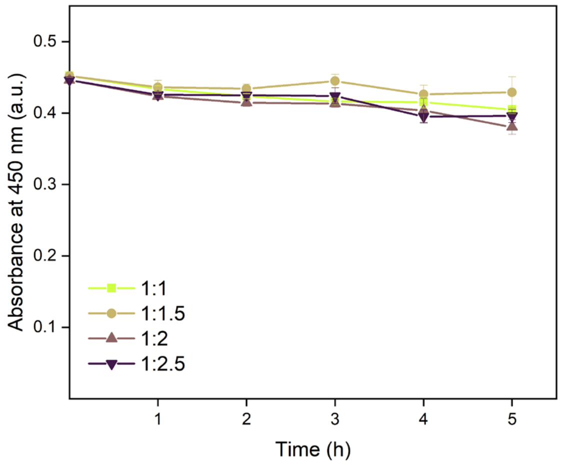

Figure S14. The absorbance change vs time of blanks at different PDADMAC:NaPSS ratios.

Micrococcus lysodeikticus assay of AL0.3 C0.3

To examine whether lysozyme can be taken up by only swelling, 0.3 M KBr was used for all steps to allow swelling without pore formation. The resulted samples did show slightly better results than blanks, but less than most R2 samples. More importantly, after salt treatment, there was no increase in activity, indicating that only limited lysozyme was captured by swelling.

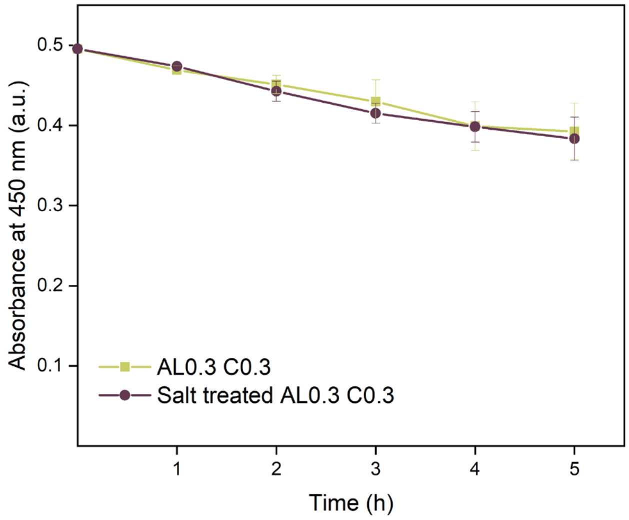

Figure S15. The absorbance change vs time of AL0.3 C0.3 samples. With the aid of salt to open up the structure, there was not much difference in the reduction of absorbance.

Micrococcus lysodeikticus assay at room temperature and 45 °C

The antibacterial property of blank saloplastics at a ratio of 1:1 and lysozyme free solution (5 mg/L) were measured at room temperature and 45 °C (Figure S16a). Heating at this temperature showed no effect on pure substrate in PBS solution. For free lysozyme solution, the activity was increased that faster decrease in absorbance was observed. However, for the blank samples, a leakage of PEs/PECs occurred when increasing the temperature. Instead of killing the bacteria, a coagulating effect was observed that bigger aggregates appeared which also caused an absorbance decrease (Figure S16a and 16b).

a)

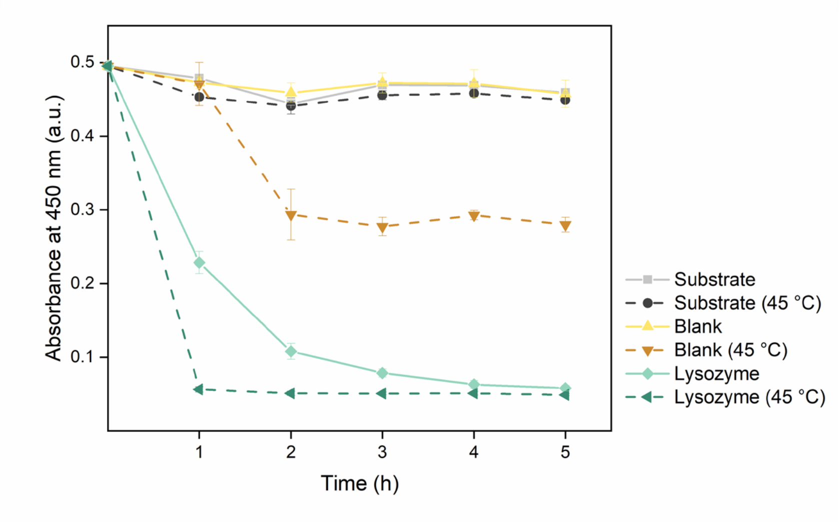

b)

Blank at rt

Blank at 45 °C

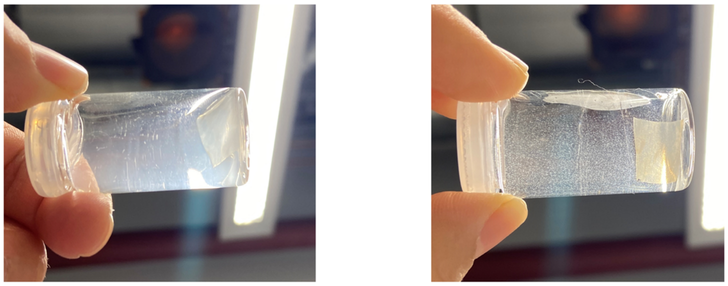

Figure S16. a) The absorbance change vs time of blank saloplastics and free lysozyme solution at room temperature and 45 °C. b) Bigger aggregates of bacteria were formed probably caused by leaching of PEs/PECs.

Micrococcus lysodeikticus assay of samples at different ratios

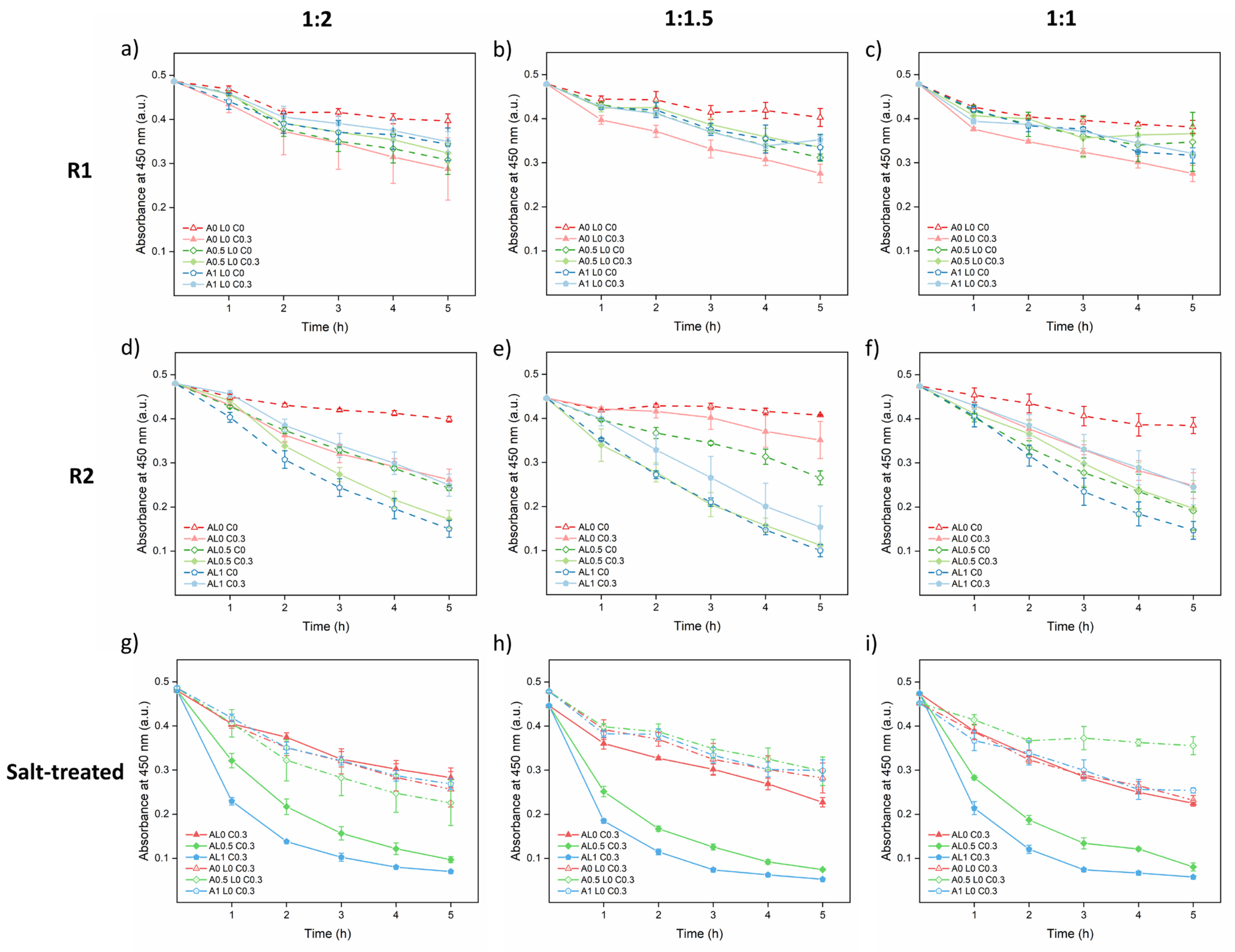

Figure S17. The absorbance change vs time of samples at different PDADMAC:NaPSS ratios.
